# Global epidemiology of retinal vein occlusion: a systematic review and meta-analysis of prevalence, incidence, and risk factors

**DOI:** 10.7189/jogh.09.010427

**Published:** 2019-06

**Authors:** Peige Song, Yuehong Xu, Mingming Zha, Yan Zhang, Igor Rudan

**Affiliations:** 1 Centre for Global Health Research, Usher Institute of Population Health Sciences and Informatics, University of Edinburgh, Edinburgh, Scotland, UK; 2 Medical school Southeast University, Nanjing, Jiangsu, China; 3 Faculty of Life Science and Medicine, Kings College London, London, England, UK

## Abstract

**Background:**

Retinal vein occlusion (RVO) is the second most common retinal vascular disorder that affected 16.4 million people worldwide in 2008. The last decade has seen new epidemiological data on RVO, enabling us to provide a contemporary estimation of RVO epidemiology.

**Methods:**

We searched PubMed, Medline, Embase, GLOBAL HEALTH, World Health Organization Global Health Library, China National Knowledge Infrastructure for studies that reported prevalence or incidence of RVO in the general population. The age- and sex-specific prevalence of RVO was estimated by a multilevel mixed-effects logistic regression, the incidence of RVO and potential risk factors for RVO were respectively pooled by a random-effects meta-analysis.

**Results:**

The prevalence of any RVO, branch RVO (BRVO) and central RVO (CRVO) all increased with advanced age, but didn’t differ significantly between sexes. In 2015, the global prevalence of any RVO, BRVO and CRVO in people aged 30-89 years was 0.77% (95% confidence interval CI = 0.55-1.08), 0.64% (95% CI = 0.47-0.87) and 0.13% (95% CI = 0.08-0.21), equivalent to an overall of 28.06 million, 23.38 million and 4.67 million affected people. For any RVO, the pooled five-year cumulative incidence was 0.86% (95% CI = 0.70-1.07) and the pooled ten-year cumulative incidence was 1.63% (95% CI = 1.38-1.92). Hypertension was the strongest risk factor for any RVO, with a meta- odds ratio (OR) of 2.82 (95% CI = 2.12-3.75).

**Conclusions:**

This study provides an updated summary of RVO epidemiology in the general population. More epidemiological studies worldwide are still needed to better understand the global disease burden of RVO.

Retinal vein occlusion (RVO), an obstruction of normal venous system of the retina, is an important cause of visual loss and visual handicap [1,2]. According to the site of occlusion, RVO can be broadly classified as branch RVO (BRVO) and central RVO (CRVO). BRVO typically occurs at an arteriovenous intersection, whereas CRVO at or near the lamina cribrosa of the optic nerve [2-5]. Taken individually, BRVO is more prevalent, but less visually damaging, than CRVO [4,6-8]. Although people with RVO are generally asymptotic and painless at the early stages, variable (or even severe) vision loss could be resulted in by complications of RVO, especially that of CRVO [1,9,10]. Common complications of BRVO and CRVO include macular edema, macular ischemia, which are persistent and difficult to treat [1,5].

Previous studies have suggested RVO to be the second most common retinal vascular disorder following diabetic retinopathy [1,3,4]. Unfortunately, the research attention on RVO is rather less compared with abundant studies on diabetic retinopathy, especially that on epidemiology. Epidemiology of RVO serves as the basis for public health resource allocation and policy making [1,6]. In 2010, Rogers S and colleagues made the first effort to estimate the prevalence of RVO worldwide (Global RVO Study 2010) [6]. According to their estimates, 16.4 million people aged 30 years and above were suffering from RVO in 2008, among whom 13.9 million were with BRVO and 2.5 million with CRVO [6]. Previous studies have documented advanced age, hypertension and other vascular factors as risk factors for RVO [11-13]. With global ageing trend and the expanding burden of cardiovascular diseases, it is expected that RVO might place an increasing burden on society. Since the release of Global RVO Study 2010, several new (national and sub-national) investigations on the prevalence of RVO have been published, it is, therefore, necessary to update the previous estimation. Moreover, the incidence of RVO has been systematically reported but never been synthesized in a meta-analysis manner [1].

To fill the gaps in the evidence matrix, we conducted a systematic review to retrieve epidemiological studies that reported the prevalence or incidence of RVO. The aims of this study are as follows: (1) to estimate the prevalence of RVO in the general population; (2) to generate the global number of people with RVO in 2015; (3) to pool the overall incidence of RVO in the general population; and (4) to evaluate the effects of potential risk factors for RVO.

## METHODS

This systematic review was conducted and reported in accordance with the Preferred Reporting Items for Systematic reviews and Meta-Analyses (PRISMA) guidelines and the Guidelines for Accurate and Transparent Health Estimates Reporting (GATHER) statement [14,15].

### Search strategy and selection criteria

To identify all published studies that reported epidemiology of RVO in the general population, we conducted a systematic literature search in six bibliographic databases, namely, PubMed, Medline, Embase, GLOBAL HEALTH, World Health Organization Global Health Library, China National Knowledge Infrastructure up to June 6, 2018. The search terms were combinations of epidemiology (prevalence, incidence or epidemiology) and RVO (retinal vein occlusion, retinal vein obstruction), in forms of free words or controlled vocabulary (ie, medical subject headings). No time or language limitations were placed on searches or returned results. The specific search strategies for each bibliographic database are listed in Table S1 in **Online Supplementary Document**. The reference lists of all the included studies were further scrutinised to locate potentially relevant studies that had been omitted.

Two researchers (YX and MZ) independently examined all returned citations in two stages: screening of titles and abstracts and full-text appraisal. In this study, we only included studies that reported prevalence or incidence of RVO in the general population. Studies that were conducted in a specifically selected group that could not represent the general population (eg, hypertensive patients, people with other cardiovascular diseases) or in clinical settings were excluded to avoid potential bias in representativeness of the general population. The included studies should have reported the prevalence or incidence of RVO based on the number of individuals affected by RVO, rather than the number of eyes with RVO. Furthermore, studies were excluded if RVO was not assessed by fundus photography. In case of multiple publications based on the same investigation, we compared those publications and kept the one with the most comprehensive results.

### Data extraction and quality assessment

In the context of this study, any RVO was predefined to include both BRVO and CRVO. Relevant information, including study characteristics (author[s], year of publication, study setting, year of investigation, study design, sampling method, assessment and diagnosis of RVO) and data on prevalence (sample size and number of cases) or incidence (sample at risk and number of new cases), was extracted from the included studies by two researchers (YX and MZ). Whereas the prevalence or incidence was reported by age or sex groups within the same study, data were correspondingly split into different groups. For studies where censoring age groups (eg, less than 40 years or older than 70 years) were employed, we imputed the missing age band by taking the same width in other complete age groups within the same study. A subset of studies reported the risk factors for RVO, we chose those based on multivariate study design and extracted estimates of odds ratios (ORs) and corresponding 95% confidence intervals (CIs).

The quality of included studies was assessed by using the Strengthening the Reporting of Observational Studies in Epidemiology (STROBE) guideline [16]. The assessment included five modules, namely, sample population, sample size, participation rate, outcome assessment, and analytical methods. Each module was graded as with high risk and unclear (score 0), moderate risk (score 1) or low risk (score 2) (see Table S2 in **Online Supplementary Document**). The overall bias risk of each study was represented by the total score of the five modules. All disagreements in the review stage and data extraction process were resolved by consensus through discussion.

### Statistical analysis

#### Meta-regression of the prevalence of any RVO, BRVO and CRVO

Before pooling prevalence estimates of RVO, we first assessed the heterogeneity among studies using the Cochran's Q statistic and *I*^2^ index (the proportion of total variability due to true between-study heterogeneity beyond chance) [17-19]. The influence of a single study was checked by a leave-one-out sensitivity analysis [20,21]. We also examined publication bias by visual inspection of funnel plots, Egger's regression test for funnel plot asymmetry and Begg’s rank correlation test [22-24]. A random-effects meta-analysis was employed a priori throughout this study because of inherent variations between study characteristics (eg, investigated sample, study design and study location).

In the data extraction process, some studies reported prevalence estimates in stratified groups of age or sex. To investigate potential sources of heterogeneity between studies reporting the prevalence of RVO, as well as to take into this hierarchical data structure into consideration, a multilevel mixed-effect meta-regression was conducted [25,26]. This was done for any RVO, BRVO and CRVO respectively.

#### Estimation of the number of people with any RVO, BRVO and CRVO

To estimate the number of people living with any RVO, BRVO and CRVO, the age- and sex-specific prevalence generated from meta-regression was applied to the corresponding age- and sex-specific population size, available from the United Nations Population Division (UNPD) [27]. This was done for the year 2015 and in people aged 30-89 years.

#### Meta-analysis of the incidence of and risk factors for any RVO

A limited number of studies reported the incidence of RVO, ie, five-year cumulative incidence, nine-year cumulative incidence, ten-year cumulative incidence and 15-year cumulative incidence. As a rule, at least three data points should be available in the meta-analysis. Therefore, we only pooled five-year cumulative incidence and ten-year cumulative incidence for any RVO by a random-effects meta-analysis.

A subset of studies that reported the prevalence of RVO also reported potential risk factors for RVO based on multivariate study design. We selected the factors with at least three data points and similar definitions. Finally, nine risk factors for any RVO, namely, advanced age (per decade increase), female sex, creatinine (per 10 mmol/L increase), vertical cup-to-disc ratio (per 1.0 increase), heart attack, total cholesterol (per mmol/L increase), diabetes, stroke and hypertension, were kept for the subsequent random-effects meta-analyses.

All statistical analyses were conducted with R version 3.3.0 (R Foundation for Statistical Computing, Vienna, Austria) and STATA version 14.0 (STATA Corporation, College Station, Texas, USA). A *P*-value of less than 0.05 indicated statistical significance.

## RESULTS

### Summary of the systematic review

**Figure 1:**
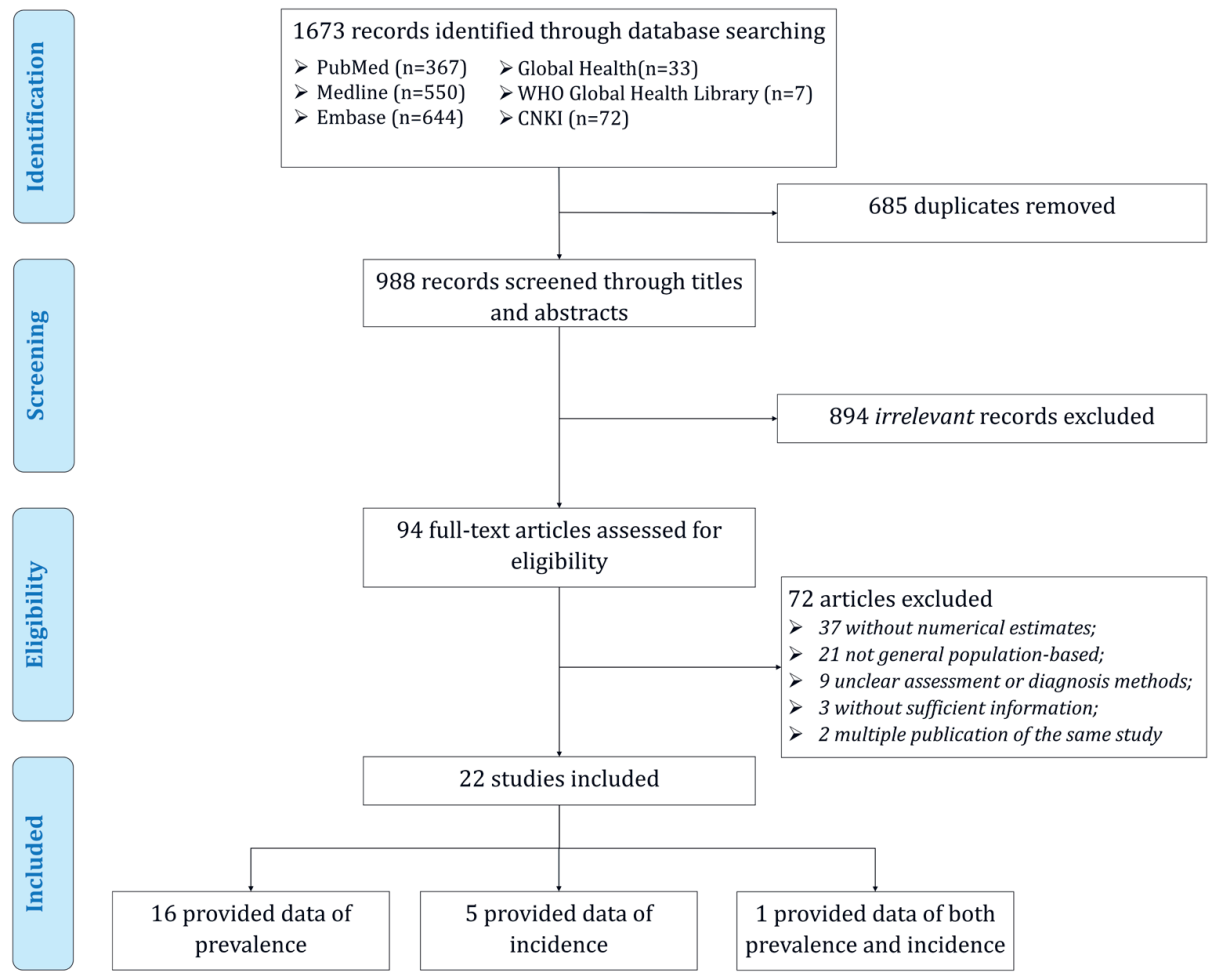
Flow diagram of systematic review.

As shown in **Figure 1**, our initial literature search returned 1673 citations from six bibliographic databases. After removing 685 duplicate records, we assessed 988 records for relevance by titles and abstracts. Then 94 records were reviewed in full-text, among which 22 met the selection criteria and were included in this systematic review. Of the 22 eligible studies, 16 provided prevalence data, five provided incidence data, and one provided both prevalence and incidence data. A summary of the main characteristics of all included studies is demonstrated in **Table 1** and the detailed characteristics of each included study can be found in Table S3 in **Online Supplementary Document**. All the included studies employed fundus imaging to assess the presence of RVO. A total of 17 studies provided data on RVO prevalence, where a total of 1056 patients were with RVO out of 120771 participants. More than half (n = 11, 64.7%) of the 17 studies were published from 2010 onwards, with the majority (n = 12, 70.6%) being conducted in mixed settings and more than half (n = 9, 52.9%) in Western Pacific Region. More than half (n = 10, 58.8%) of those studies provided prevalence for both males and females and mostly were with more than 4000 participants (n = 12, 70.6%). For studies reporting the incidence of RVO, the majority (n = 5, 83.3%) were published between 2000 and 2009, conducted in urban settings and with a sample size of 2001–4000. Moreover, half of the six studies on RVO incidence provided data on both males and females. Those studies were either in Region of the Americas (n = 3, 50%) or Western Pacific Region (n = 3, 50%). All the included studies were with a quality score of at least 6 (see Table S4 in **Online Supplementary Document** for more details).

**Table 1:** Main characteristics of the included studies on RVO epidemiology (n = 22)

Characteristic	Number of studies (%)	
**Studies on RVO prevalence (n = 17)**	**Studies on RVO incidence (n = 6)**
**Year published:**		
1990-1999	1 (5.9)	0 (0.0)
2000-2009	5 (29.4)	5 (83.3)
2010-2018	11 (64.7)	1 (16.7)
**Setting:**		
Urban	2 (11.8)	5 (83.3)
Rural	3 (17.6)	0 (0.0)
Mixed	12 (70.6)	1 (16.7)
**Sex:**		
Both	10 (58.8)	3 (50.0)
Mixed	7 (41.2)	3 (50.0)
Sample size:*		
<2000	2 (11.8)	1 (16.7)
2001–4000	3 (17.6)	5 (83.3)
4001–6000	5 (29.4)	0 (0.0)
6001–10000	4 (23.5)	0 (0.0)
>10000	3 (17.6)	0 (0.0)
**Region:**		
African Region	0 (0.0)	0 (0.0)
Region of the Americas	4 (23.5)	3 (50.0)
European Region	2 (11.8)	0 (0.0)
South-East Asia Region	2 (11.8)	0 (0.0)
Western Pacific Region	9 (52.9)	3 (50.0)
Eastern Mediterranean Region	0 (0.0)	0 (0.0)
**Quality score:**		
10	1 (5.9)	0 (0.0)
9	4 (23.5)	3 (50.0)
8	5 (29.4)	2 (33.3)
7	5 (29.4)	1 (16.7)
6	2 (11.8)	0 (0.0)

RVO – retinal vein occlusion

*One study provided both prevalence and incidence data, therefore the sum of studies on RVO prevalence and studies on RVO incidence (n = 23) exceeded the total number of included studies (n = 22);* Sample size referred to sample at risk for studies reporting RVO incidence.

### Age- and sex-specific prevalence of any RVO, BRVO and CRVO

**Figure 2:**
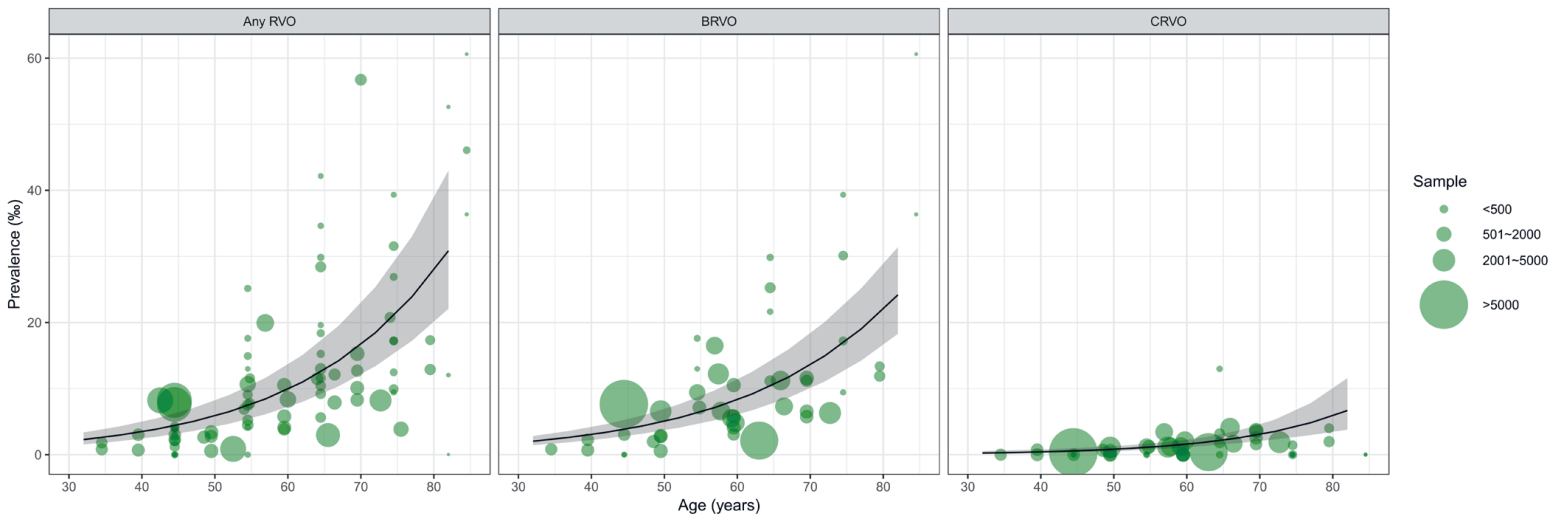
The relation between age and prevalence of any retinal vein occlusion (RVO), branch RVO (BRVO) and central RVO (CRVO) based on informative data points from the included studies. Note: The bubbles are informative data points from included studies that reported the prevalence of RVO; the size of each bubble is proportional to the sample size.

Substantial heterogeneity was noted among individual studies that reported prevalence of any RVO (*I*^2^ = 93.6%, *P* < 0.001), BRVO (*I*^2^ = 93.1%, *P* < 0.001) and CRVO (*I*^2^ = 71.8%, *P* < 0.001) respectively. However, no single study was found to have had disproportionally excessive influence on the pooled results and no publication bias was detected (see Tables S5-S7 in **Online Supplementary Document** for more details). A substantial number of data points were available to construct the relation of prevalence and age (**Figure 2**). Across the age spectrum from the early 30s to late 80s, the prevalence of any RVO and BRVO both increased steadily with advanced age. For CRVO, this positive relationship between prevalence and age was less pronounced.

Based on the multilevel mixed-effect meta-regression, we estimated the age- and sex-specific prevalence of any RVO, BRVO and CRVO respectively (**Table 2**). The prevalence estimates did not differ significantly between sexes. Overall, the prevalence of BRVO ranged from 0.23% (95% CI = 0.17-0.32) in people aged 30-39 years to 2.64% (95% CI = 2.00-3.39) in those aged 80-89 years, and that of CRVO ranged from 0.03% (95% CI = 0.01-0.06) to 0.75% (95% CI = 0.41-1.35). For any RVO, the prevalence increased from 0.26% (95% CI = 0.18-0.38) in individuals aged 30-39 years to 3.39% (95% CI = 2.42-4.74) in those aged 80-89 years. Taken collectively, the overall prevalence estimates of any RVO, BRVO and CRVO in people aged 30-89 years in 2015 were 0.77% (95% CI = 0.55-1.08), 0.64% (95% CI = 0.47-0.87) and 0.13% (95% CI = 0.08-0.21) respectively.

**Table 2:** Estimated age- and sex-specific prevalence of any RVO, BRVO and CRVO*

Age group (years)	Prevalence of any RVO (%)		Prevalence of BRVO (%)		Prevalence of CRVO (%)	
**Male**	**Female**	**Male**	**Female**	**Male**	**Female**
30-39	0.26	0.26	0.23	0.23	0.03	0.03
	(0.18-0.38)	(0.18-0.38)	(0.16-0.32)	(0.17-0.32)	(0.01-0.06)	(0.01-0.06)
40-49	0.44	0.44	0.38	0.39	0.06	0.06
	(0.31-0.62)	(0.32-0.63)	(0.28-0.52)	(0.28-0.53)	(0.03-0.10)	(0.03-0.10)
50-59	0.74	0.74	0.63	0.63	0.11	0.11
	(0.53-1.02)	(0.54-1.03)	(0.46-0.86)	(0.47-0.86)	(0.08-0.16)	(0.07-0.16)
60-69	1.23	1.24	1.02	1.03	0.21	0.21
	(0.90-1.69)	(0.91-1.71)	(0.75-1.39)	(0.76-1.41)	(0.15-0.30)	(0.15-0.30)
70-79	2.06	2.09	1.66	1.69	0.41	0.41
	(1.50-2.84)	(1.51-2.88)	(1.24-2.20)	(1.25-2.25)	(0.26-0.64)	(0.26-0.63)
80-89	3.36	3.41	2.60	2.66	0.75	0.75
	(2.40-4.68)	(2.43-4.78)	(1.98-3.34)	(2.02-3.43)	(0.41-1.35)	(0.41-1.34)
Overall (30-89)	0.74	0.81	0.64	0.65	0.13	0.13
	(0.53-1.03)	(0.58-1.12)	(0.47-0.86)	(0.48-0.88)	(0.08-0.20)	(0.08-0.21)

RVO – retinal vein occlusion, BRVO – branch retinal vein occlusion, CRVO – central retinal vein occlusion

*The overall prevalence of RVO, BRVO and CRVO was adjusted to the demographic structure in 2015.

### Global number of people with any RVO, BRVO and CRVO in 2015

By multiplying the estimated age- and sex-specific prevalence of RVO with the corresponding demographic data, the number of people with RVO in 2015 was generated (**Table 3**). Globally, there were 28.06 million (95% CI = 20.12-39.07) people living with any RVO in 2015, among whom 83.3% (23.38 million [95% CI = 17.20-31.54]) were with BRVO and 16.7% (4.67 million [95% CI = 2.92-7.52]) were with CRVO. The age group that contributed the most cases was 60-69 years for any RVO and BRVO, and 70-79 years for CRVO.

**Table 3:** Estimated age- and sex-specific number of people with any RVO, BRVO and CRVO in 2015

Age group (years)	People with any RVO (million)			People with BRVO (million)			People with CRVO (million)		
**Male**	**Female**	**Overall**	**Male**	**Female**	**Overall**	**Male**	**Female**	**Overall**
30-39	1.39	1.36	2.75	1.23	1.21	2.44	0.16	0.15	0.31
	(0.95-2.03)	(0.94-1.98)	(1.89-4.01)	(0.88-1.70)	(0.87-1.66)	(1.75-3.36)	(0.08-0.33)	(0.07-0.32)	(0.14-0.65)
40-49	2.09	2.07	4.17	1.82	1.81	3.63	0.27	0.27	0.54
	(1.48-2.96)	(1.47-2.92)	(2.95-5.88)	(1.32-2.49)	(1.32-2.46)	(2.64-4.94)	(0.16-0.47)	(0.15-0.47)	(0.31-0.94)
50-59	2.73	2.78	5.51	2.32	2.37	4.69	0.41	0.41	0.82
	(1.97-3.78)	(2.01-3.84)	(3.99-7.62)	(1.69-3.17)	(1.74-3.23)	(3.43-6.40)	(0.28-0.61)	(0.27-0.62)	(0.55-1.22)
60-69	3.04	3.28	6.32	2.52	2.72	5.24	0.53	0.56	1.08
	(2.22-4.17)	(2.39-4.50)	(4.60-8.67)	(1.84-3.42)	(2.00-3.71)	(3.84-7.13)	(0.37-0.74)	(0.39-0.79)	(0.76-1.54)
70-79	2.53	3.07	5.60	2.03	2.47	4.50	0.50	0.60	1.10
	(1.84-3.49)	(2.22-4.23)	(4.06-7.72)	(1.52-2.70)	(1.84-3.30)	(3.36-6.00)	(0.32-0.78)	(0.38-0.93)	(0.70-1.72)
80-89	1.46	2.24	3.70	1.13	1.75	2.88	0.33	0.50	0.82
	(1.04-2.03)	(1.60-3.14)	(2.64-5.17)	(0.86-1.45)	(1.32-2.26)	(2.19-3.70)	(0.18-0.58)	(0.27-0.88)	(0.45-1.47)
Total (30-89)	13.25	14.81	28.06	11.41	11.97	23.38	2.27	2.40	4.67
	(9.50-18.45)	(10.62-20.61)	(20.12-39.07)	(8.38-15.43)	(8.83-16.11)	(17.20-31.54)	(1.43-3.63)	(1.49-3.89)	(2.92-7.52)

RVO – retinal vein occlusion, BRVO – branch retinal vein occlusion, CRVO – central retinal vein occlusion

### Pooled five-year and ten-year cumulative incidence of any RVO

**Figure 3:**
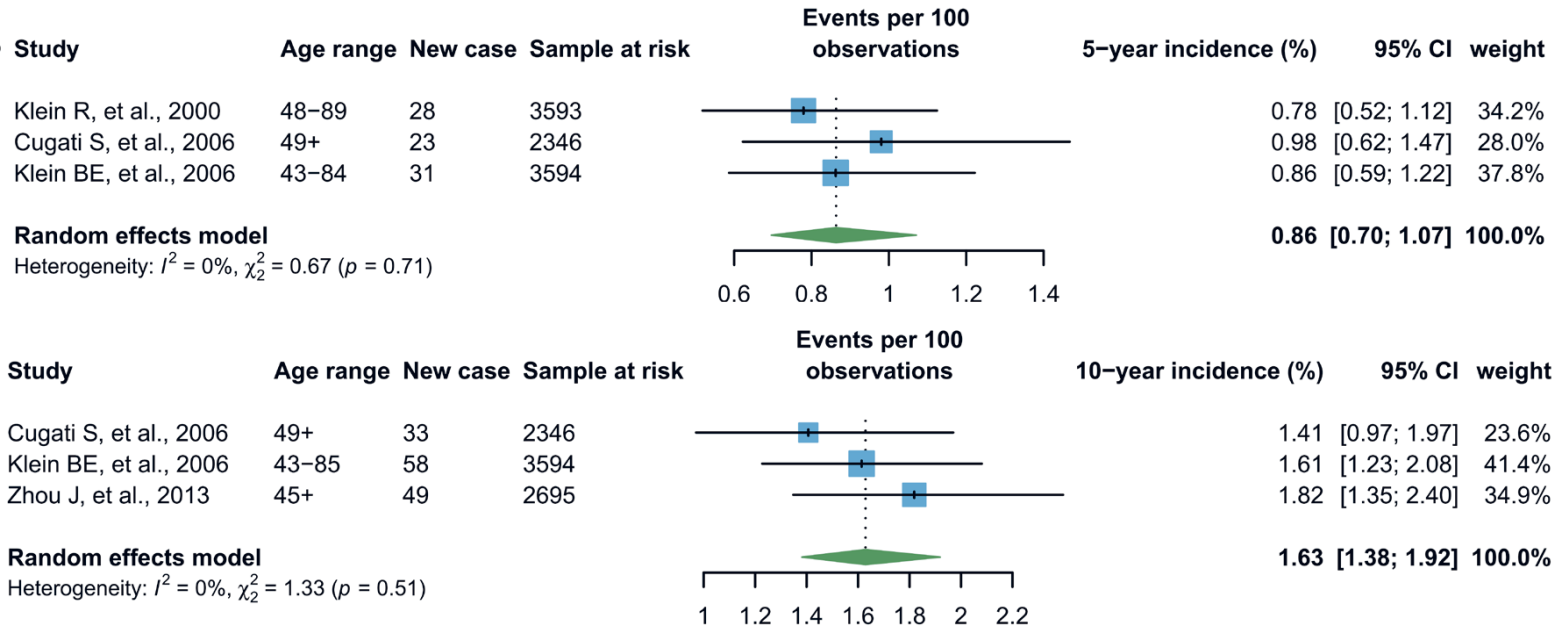
Pooled five-year and ten-year cumulative incidence of any retinal vein occlusion (RVO).

Six individual studies reported four forms of incidence of any RVO, namely, five-year, nine-year, ten-year and 15-year cumulative incidence. Among those, five-year and ten-year cumulative incidence was respectively reported in three individual studies and therefore were synthesized by a random-effects meta-analysis. As shown in **Figure 3**, the pooled five-year cumulative incidence was 0.86% (95% CI = 0.70-1.07) and the pooled ten-year cumulative incidence was 1.63% (95% CI = 1.38-1.92). Additionally, the Hisayama Study reported a nine-year cumulative incidence of any RVO as 3.0% in Japan [28], and the Beaver Dam Eye Study reported a 15-year cumulative incidence of any RVO as 2.3% in the United States of America (USA) [29].

### Synthesized effect size of risk factors for any RVO

A total of 12 individual studies that reported the prevalence of RVO additionally examined potential risk factors for RVO using multivariate design. Nine risk factors for any RVO were with at least three contributing data points and therefore were included for synthesis (**Table 4**). In line with our age- and sex-specific prevalence of any RVO by meta-regression, advanced age was found to be a significant risk factor for any RVO, with a meta-OR of 1.60 (95% CI = 1.38-1.84) for every decade increase, whereas sex was found to have no influence on the prevalence of any RVO. Hypertension was revealed as the strongest risk factor for any RVO (meta-OR: 2.82 [95% CI = 2.12-3.75]), followed by heart attack history (meta-OR: 2.23 [95% CI = 1.09-4.55]) and stroke history (meta-OR: 2.07 [95% CI = 1.30-3.29]). In addition, higher levels of total cholesterol and creatinine were also with higher risk of any RVO, with meta-ORs of 1.32 (95% CI = 1.08-1.61) for every mmol/L increase and 1.04 (95% CI = 1.02-1.07) for every ten-mmol/L increase respectively.

**Table 4:** Synthesized effect size of nine risk factors for any RVO

Risk factor	Number of data points	OR (95% CI)	z value	*P*-value
Risk factor 1-Advanced age (per decade increase)	9	1.60 (1.38-1.84)	6.36	<0.001
Risk factor 2-Female sex	6	1.08 (0.76-1.55)	0.44	0.661
Risk factor 3-Creatinine (per 10 mmol/L increase)	3	1.04 (1.02-1.07)	3.06	0.002
Risk factor 4-Vertical cup-to-disc ratio (per 1.0 increase)	3	4.87 (0.88-27.04)	1.81	0.070
Risk factor 5-Heart attack	3	2.23 (1.09-4.55)	2.19	0.028
Risk factor 6-Total cholesterol (per mmol/L increase)	3	1.32 (1.08-1.61)	2.66	0.008
Risk factor 7-Diabetes	4	1.19 (0.52-2.70)	0.42	0.677
Risk factor 8-Stroke	4	2.07 (1.30-3.29)	3.08	0.002
Risk factor 9-Hypertension	9	2.82 (2.12-3.75)	7.11	<0.001

CI – confidence interval, RVO – retinal vein occlusion

## DISCUSSION

This systematic review portrays a comprehensive picture of the global epidemiology of RVO based on published evidence on prevalence, incidence and risk factors. The data-driven estimates of RVO prevalence in different age and sex groups were presented, where the prevalence estimates increased with advanced age but did not differ significantly between sexes. In 2015, the global prevalence of any RVO, BRVO and CRVO in people aged 30-89 years was 0.77%, 0.64% and 0.13%, translating to a total of 28.06 million, 23.38 million and 4.67 million affected individuals respectively. For any RVO, the pooled five-year cumulative incidence was 0.86% and the pooled ten-year cumulative incidence was 1.63%. Except for advanced age, another five risk factors for any RVO were confirmed, which were hypertension, heart attack history, stroke history, higher level of total cholesterol and higher level of creatinine.

Subsequent to the Global RVO Study 2010 by Rogers S and colleagues, our study made the second attempt of estimating the global prevalence of RVO [6]. By far, this study provides the most up-to-date estimation of RVO prevalence and cases at the global level and summarizes the incidence of and risk factors for RVO in the general population for the first time. Benefiting from a comprehensive systematic review process, a dual review process and rigorous selection criteria, our ability to capture all published studies on RVO epidemiology has been well guaranteed. For estimating the prevalence of RVO, a total of 17 individual studies were ultimately included for meta-regression. Among those studies, almost half (eight studies) were published after the year 2010 when the Global RVO Study 2010 was published, implying the necessity of our updated analysis [6]. Furthermore, we only included studies that were conducted in the general population, therefore the generalizability of our estimation on RVO epidemiology could be largely ensured. Regarding potential risk factors for RVO, we only included studies that were based on a multivariate design to reduce suspected bias inherent to the univariate study design [30].

Despite the abovementioned strengths, this study is not free from limitations. First, 17 individual studies contributed prevalence data of RVO in meta-regression, although age- and sex-specific prevalence estimates of RVO was successfully generated based on data points from those studies, the estimation was only for the global level. The limited number of studies restricted our ability to conduct any regional analysis. In fact, all the data points for generating the prevalence of RVO came from four regions, namely, Region of the Americas, European Region, South-East Asia Region and Western Pacific Region. The lack of contributing information from African Region and Eastern Mediterranean Region might have caused an overestimation of RVO prevalence in our study, given hypertension and other cardiovascular diseases are positively linked to the presence of RVO. However, that was for not sure because no comparisons between the prevalence of RVO in African Region/Eastern Mediterranean Region and that in other regions have been made before. Second, for the incidence of RVO, we were only able to pool the five-year and ten-year cumulative incidence of any RVO. Moreover, these two synthesized incidence estimates were both based on three contributing data points from the USA, Australia and China, therefore their generalizability is not optimistic even at the global level. Third, for potential risk factors for RVO, we only examined the effects of nine risk factors, among which six were revealed as statistically significant. Because of the paucity of data sources, the effects of other risk factors, including carotid artery disease, smoking, etc., were not assessed in the current study [1,11,12,31]. With new epidemiological data coming in, those shortcomings might be overcome in the foreseeable future.

In this study, RVO, including any RVO, BRVO and CRVO, was found to be highly age-driven. RVO is a vascular disease of the retina, its degenerative nature has become a common notion of this disease, and the increasing trend of prevalence associated with advanced age has also been widely observed in previous epidemiological studies [6,32,33]. Our prevalence estimates of any RVO (0.77%), BRVO (0.64%) and CRVO (0.13%) in 2015 were all higher than those in the Global RVO Study 2010, which reported an overall prevalence of 0.52%, 0.44% and 0.08% for any RVO, BRVO and CRVO in 2008 respectively [6]. Given the fact that RVO prevalence rises with advanced age and the global demographic ageing process, it is plausible to note an increasing trend in the overall prevalence of RVO during 2008-2015. In view of the incidence of RVO, the five-year cumulative incidence of any RVO was pooled as 0.86%, and the ten-year cumulative incidence was almost doubled as 1.63%. This upward trend of cumulative incidence could also be regarded as a reflection of the cumulative effect of arteriosclerosis and vascular ageing [6,31].

In line with the Global RVO Study 2010 and many previous epidemiological investigations, our prevalence estimates of RVO didn’t vary significantly between sexes, and this phenomenon has been observed in both the meta-regression and meta-analysis of risk factors [6,33,34]. Hypertension, a well-recognized covariate for RVO, was found to be the strongest risk factor for any RVO in our meta-analysis [31,35]. This strong and consistent link between hypertension and RVO suggests the benefits of blood pressure management in the prevention of RVO [35]. Due to a similar etiological pathway, RVO was also associated with the presence of other cardiovascular diseases in our meta-analyses, such as stroke and heart attack.

To conclude, our contemporary systematic review and meta-analysis offers a comprehensive summary of RVO epidemiology (prevalence, incidence and risk factors) in the general population. RVO is generally more common in elderly, hypertensive patients, and people with cardiovascular diseases. With the global ageing trend, the prevalence and burden of RVO are also likely to expand. More epidemiological studies, especially those on RVO incidence and in African Region and Eastern Mediterranean Region, are still needed to better understand the disease burden of RVO worldwide.

## Additional material

Online Supplementary Document
