# Supplementary material for: Global epidemiology of retinal vein occlusion: a systematic review and meta-analysis of prevalence, incidence, and risk factors
Source: J Glob Health. 2019 May 12;9(1):010427. doi: 10.7189/jogh.09.010427 (PMC6513508; doi:10.7189/jogh.09.010427)
Supplement: Online Supplementary Document [file jogh-09-010427-s001.pdf]

J Global Health 2019; 9: 010427

Global epidemiology of retinal vein occlusion: a systematic review and meta-analysis of prevalence, incidence, and risk factors

Peige Song [1], Yuehong Xu [2], Mingming Zha [2], Yan Zhang [3], Igor Rudan [1]

[1] Centre for Global Health Research, Usher Institute of Population Health Sciences and Informatics, University of Edinburgh, Edinburgh, Scotland, UK

[2] Medical school Southeast University, Nanjing, Jiangsu, China

[3] Faculty of Life Science and Medicine, Kings College London, London, England, UK

Supplementary contents

Table S1. Search strategies in bibliographic databases

Table S2. Quality score scale for assessing the risk of bias

Table S3. Characteristics of the included studies (n=22)

Table S4. Quality scores for assessing the risk of bias in the included studies (n=22)

Table S5. Meta-analyses of studies on any RVO prevalence

Table S6. Meta-analyses of studies on any BRVO prevalence

Table S7. Meta-analyses of studies on any CRVO prevalence

Table S8. Meta-analyses of risk factors for any RVO

Table S1. Search strategies in bibliographic databases

Database	Access date	Search terms
PubMed	06/06/2018	(retinal vein occlusion[Title/Abstract] OR retinal vein obstruction[Title/Abstract]) AND (prevalence[Title/Abstract] OR incidence[Title/Abstract] OR epidemiology[Title/Abstract])
Medline	06/06/2018	1 retinal vein occlusion.mp. or exp Retinal Vein Occlusion/<br>2 retinal vein obstruction.mp.<br>3 exp PREVALENCE/ or prevalence.mp.<br>4 incidence.mp. or exp INCIDENCE/<br>5 exp EPIDEMIOLOGY/ or epidemiology.mp.<br>6 1 or 2<br>7 3 or 4 or 5<br>8 6 and 7<br>9 limit 8 to humans
Embase	06/06/2018	1 retinal vein occlusion.mp. or exp retina vein occlusion/<br>2 retinal vein obstruction.mp.<br>3 prevalence.mp. or exp prevalence/<br>4 exp incidence/ or incidence.mp.<br>5 exp epidemiology/ or epidemiology.mp.<br>6 1 or 2<br>7 3 or 4 or 5<br>8 6 and 7<br>9 limit 8 to (human and embase and (article or article in press or reports or short survey))
GLOBAL HEALTH	06/06/2018	1 exp retinal vein occlusion/ or retinal vein occlusion.mp.<br>2 retinal vein obstruction.mp.<br>3 (disease incidence or epidemiology or disease prevalence).sh.<br>4 1 or 2<br>5 3 and 4
Global Health Library	06/06/2018	tw:((retinal vein occlusion OR retinal vein obstruction) AND (prevalence OR incidence OR epidemiology))<br>Index<br>LILACS (Americas) (remover)<br>WPRIM (Western Pacific) (remover)<br>IMSEAR (South-EastAsia) (remover)<br>IMEMR (Eastern Mediterranean) (remover)<br>AIM (Africa) (remover)<br>Limits<br>Adolescent (remover)<br>Child (remover)
CNKI	06/06/2018	(SU % '视网膜静脉栓塞'+'视网膜静脉阻塞'+'视网膜静脉闭塞') AND (SU % '发病率' + '发生率' + '患病率'+ '罹患率' + '现患率'+ '流行' + '调查')<br>In Medicine & Public Health category

Table S2. Quality score scale for assessing the risk of bias

Bias type	Low risk (score=2)	Moderate risk (score=1)	High risk (score=0)
Selection (sample population)	1) Sample from general population, not a select group;<br>2) Consecutive unselected population;<br>3) Rationale for case and control selection explained.	1) Sample selected from large population but selection criteria not defined;<br>2) Sample selection ambiguous but may be representative;<br>3) Rationale for cases and controls not explained;<br>4) Eligibility criteria not explained;<br>5) Analysis to adjust for sampling strategy bias.	1) Highly select population making it difficult to generalise finding;<br>2) Sample selection ambiguous and sample unlikely to be representative.
Selection (sample size)	1) Sample size calculation performed and adequate.	1) Sample size calculation performed and reasons for not meeting sample size given;<br>2) Sample size calculation not performed but all eligible persons studied.	1) Sample size estimation unclear or only sub-sample studied.
Selection (participation rate)	1) High response rate (>85%).	1) Moderate response rate (70–85%).	1) Low response rate (<70%);<br>2) Response rate not reported.
Performance bias (outcome assessment)	1) Diagnosis using consistent criteria and direct examination.	1) Assessment from administrative database or register;<br>2) Assessment from hospital record or interviewer.	1) Assessment from non-validated data or generic estimate from the overall population.
Performance bias (analytical methods to control for bias)	1) Analysis appropriate for the type of sample (subgroup analysis/regression etc.).	1) Analysis does not account for common adjustment.	1) Data confusing.

Table S3. Characteristics of the included studies (n=22)

ID	Author	Year Published	Country	Study setting	URBAN/ RURAL/ MIXED	Ethnicity	Investigation Date	Study Name	Study design	Sampling Strategy	Grading system	Outcome	MV analyses on risk factors	Sample size	Cases	Age range	Female proportion	Age-specific estimate	Sex-specific estimate
P1[1]	Mitchell P, et al.	1996	Australia	2 postcode areas in the Blue Mountains region	Urban	NS	Jan 1992-Jan 1994	The Blue Mountains Eye Study	Cross-sectional	Random sampling	NS, graded by an ophthalmologist	Prevalence	Yes	3654	59	49+	0.5677	Yes	No
P2[2]	Klein R, et al.	2000	USA	Beaver Dam, Wisconsin	Urban	99%White	Sep 1987-May 1988	The Beaver Dam Eye Study	Cross-sectional nested in a cohort study	Cluster sampling	The Wisconsin Age-Related Maculopathy grading scheme	Prevalence	Yes	4926	38	43-84	0.558	Yes	Yes
P3[3]	Wong TY, et al.	2005	USA	ARIC: 4 United States communities; CHS: Forsyth County, North Carolina and Washington County, Maryland (similar to the ARIC	Mixed	Mixed (white and black)	ARIC:1993-1995; CHS:1997-1998	The Atherosclerosis Risk in Communities & Cardiovascular Health Studies	Cross-sectional	Cluster sampling	NS, graded at the Fundus Photograph Reading Center in Wisconsin	Prevalence	Yes	15466	39	45+	0.563	Yes	No

				Study) and Sacramento County, California and Allegheny County, Pennsylvania															
P4[4]	Liu W, et al.	2007	China	Beijing	Mixed	NS	June 2001-Oct 2001	The Beijing Eye Study	Cross-sectional	Cluster sampling	NS, graded by ophthalmologists	Prevalence	No	4335	58	40+	0.56263	Yes	Yes
P5[5]	Duan Y	2008	China	Handan	Rural	NS	Oct 2006-Oct2007	The Handan Eye Study	Cross-sectional	Two-staged stratified random sampling	NS	Prevalence	Yes	6636	55	30+	0.535112	Yes	Yes
P6[6]	Cheung N, et al.	2008	USA	Six communities in the USA	Mixed	Mixed (white, black, Hispanics, Chinese)	Aug 2002-Jan2004	The Multiethnic Study of Atherosclerosis (MESA)	Cross-sectional nested in a cohort study	Random sampling	The Beaver Dam Eye Study	Prevalence	Yes	6147	65	45-84	0.523182	Yes	Yes
P7_a[7,8]	Rogers S, et al.	2010	Europe	7 centres spanning north to south Europe	Mixed	White	Dec 2000-July 2003	EUREYE Study	Cross-sectional	Cluster sampling	NS, graded at the Fundus Photograph Reading	Prevalence	No	4753	39	64-99	0.552	No	No

											Center in Wisconsin								
P7_b[7,9]	Rogers S, et al.	2010	USA	Pima and Santa Cruz counties in Arizona	Mixed	Hispanic	1997-1999	Proyecto VER Study	Cross-sectional	Random sampling of block groups	NS, graded at the Fundus Photograph Reading Center in Wisconsin	Prevalence	No	2909	58	40-96	0.612	No	No
P8[10]	Yasuda M, et al.	2010	Japan	Hisayama	Mixed	NS	1998	The Hisayama Study	Cohort	Random sampling (performed a cross-sectional examination and follow-up survey of Hisayaman residents aged 40 years or older in 1998)	NS, graded by retinal specialists	Prevalence	Yes	1775	38	40+	0.612394	Yes	Yes
P9[11]	Jonas JB, et al.	2013	India	Eight villages in Kalmeshwar Tehsil	Rural	NS	2006-2009	The Central India Eye and Medical Study (CIEMS)	Cross-sectional	Random sampling (age 30-100)	The Age-Related Eye Disease Study	Prevalence	Yes	4544	35	30-100	0.535	Yes	No
P10[12]	Wu PC, et al.	2014	China	Maqin	Rural	Chinese	Oct 2011	NS	Cross-sectional	Cluster sampling	NS	Prevalence	Yes	2511	21	40+	Not mention	Yes	No

P11[13]	Ponto KA, et al.	2015	Germany	City of Mainz or the district of Mainz-Bingen	Mixed	NS	April 2007-April 2012	the Gutenberg Health Study	Cohort	Random sample is stratified 1:1 for sex and residence (urban vs. rural) and in equal strata across four age decades	NS, graded at the Moorfields Eye Hospital Reading Center, London	Prevalence	Yes	12954	59	35-74	0.498379	Yes	Yes
P12[14]	Shin YU, et al.	2016	Korea	Korea	Mixed	NS	July 2008 - Dec 2012	KNHANES	Cross-sectional	Stratified, multistage, clustered probability sampling	NS, graded by retinal specialists	Prevalence	Yes	25765	205	19+	about 0.505	No	Yes
P13[15]	Koh V, et al.	2016	Singapore	South-western part of Singapore	Mixed	Chinese, Indian and Malay	2004-2011	The Singapore Epidemiology of Eye Disease Study: SEED ONLY SCES	Cross-sectional	Randomly selected based on an age-stratified random sampling strategy	The Blue Mountains Eye Study	Prevalence	Yes	Chinese: 3312; Indian:3337; Malay:3265	Chinese:23; Indian:26; Malay:22	40-84	Chinese:0.504; Indian:0.498; Malay:0.52	Yes	No
P14[16]	Duan X	2017	China	Qingyang	Mixed	Chinese	Jan 2014-Jan 2016	NS	Cross-sectional	Two-staged stratified random sampling	NS	Prevalence	No	7930	120	25+	0.37995	Yes	Yes
P15[17]	Thapa R, et al.	2017	Nepal	Bhaktapur district	Mixed	NS	Aug 2013 - Dec 2015	the Bhaktapur retina study	Cross-sectional	Cluster sampling	NS, graded by retinal specialists	Prevalence	No	1860	55	60-95	0.5586	Yes	Yes

P16[18]	Keel S, et al.	2018	Australia	30 sites across five Australian States and one Territory, stratified by remoteness.	Mixed	White (non-Indigenous)	March 2015 and April 2016.	The National Eye Health Survey (2015–2016)	Cross-sectional	Multi-stage, random-cluster sampling	NS, graded at the Centre for Eye Research Australia	Prevalence	Yes	non-Indigenous Australians: 3010; Indigenous Australians: 1682	non-Indigenous Australians: 27; Indigenous Australians: 14	non-Indigenous Australians: 50+; Indigenous Australians: 40+	non-Indigenous Australians: 0.589; Indigenous Australians: 0.537	Yes	Yes
I1[2]	Klein R, et al.	2000	USA	Beaver Dam, Wisconsin	Urban	99%White	Mar 1993-June 1995	The Beaver Dam Eye Study	Cross-sectional nested in a cohort study	Cluster sampling	The Wisconsin Age-Related Maculopathy grading scheme	5-year cumulative incidence	Yes	3593	28	48-89	Not mention	Yes	Yes
I2[19]	Cugati S, et al.	2006	Australia	West of Sydney	Urban	Largely white	1997-1999	The Blue Mountains Eye Study	Cohort	Cluster sampling	NS, graded by retinal specialists	5-year cumulative Incidence and 10-year cumulative Incidence	Yes	2346	23 in 5 years and 33 in 10 years	49+	0.594	Yes	No
I3[20]	Klein BE, et al.	2006	USA	Beaver Dam, Wisconsin	Urban	99%White	Mar 1998-June 2000	The Beaver Dam Eye Study	Cohort	Cluster sampling	The Wisconsin Age-Related Maculopathy	5-year cumulative Incidence and 10-year	No	3594	31 in 5 years and 58	43-84	0.563	No	No

											grading scheme	cumulative Incidence			in 10 years				
I4[21]	Arakawa S, et al.	2007	Japan	Hisayama	Urban	NS	1998	The Hisayama Study	Cohort	Cluster sampling	The Wisconsin Age-Related Maculopathy grading scheme	9-year cumulative Incidence	Yes	1369	41	49+	0.628926	Yes	Yes
I5[22]	Klein R, et al.	2008	USA	Beaver Dam, Wisconsin	Urban	99%White	Mar 2003-April 2005	The Beaver Dam Eye Study	Cross-sectional nested in a cohort study	Cluster sampling	The Wisconsin Age-Related Maculopathy grading scheme	15-year cumulative Incidence	Yes	3684	83	58-99	0.569	No	No
I6[23]	Zhou J, et al.	2013	China	Beijing	Mixed	NS	2011	The Beijing Eye Study	Cohort	Cluster sampling	NS	10-year cumulative Incidence	Yes	2695	49	45+	0.577	No	Yes

Note: NS, not specified; USA, United States of America; For studies reporting RVO incidence, sample size referred to sample at risk and cases referred to number of new cases.

Table S4. Quality scores for assessing the risk of bias in the included studies (n=22)

ID	Author	Year Published	Quality score					
			Sample population	Sample size	Partici pation	Outcome assessment	Analytical methods	Total scores
P1[1]	Mitchell P, et al.	1996	2	1	2	2	1	8
P2&I1[2]	Klein R, et al.	2000	2	2	1	2	2	9
P3[3]	Wong TY, et al.	2005	2	1	1	1	1	6
P4[4]	Liu W, et al.	2007	2	2	1	2	1	8
P5[5]	Duan Y	2008	2	2	2	2	2	10
P6[6]	Cheung N, et al.	2008	2	1	2	2	2	9
P7_a[7,8]	Rogers S, et al.	2010	2	1	1	2	1	7
P7_b[7,9]	Rogers S, et al.	2010	2	1	1	2	1	7
P8[10]	Yasuda M, et al.	2010	2	1	0	2	2	7
P9[11]	Jonas JB, et al.	2013	2	1	2	2	1	8
P10[12]	Wu PC, et al.	2014	2	2	0	2	1	7
P11[13]	Ponto KA, et al.	2015	2	1	2	2	2	9
P12[14]	Shin YU, et al.	2016	2	1	0	2	1	6
P13[15]	Koh V, et al.	2016	2	1	1	2	2	8
P14[16]	Duan X	2017	2	2	0	1	2	7
P15[17]	Thapa R, et al.	2017	2	1	2	2	1	8
P16[18]	Keel S, et al.	2018	2	1	2	2	2	9
I2[19]	Cugati S, et al.	2006	2	2	1	2	1	8
I3[20]	Klein BE, et al.	2006	2	2	1	1	1	7
I4[21]	Arakawa S, et al.	2007	2	2	1	2	2	9
I5[22]	Klein R, et al.	2008	2	2	2	2	1	9
I6[23]	Zhou J, et al.	2011	2	2	0	2	2	8

Table S5. Meta-analyses of studies on any RVO prevalence

Meta-analysis of studies that reported the prevalence of any RVO revealed significantly high heterogeneity between studies (I^2 =93.6%, p<0.001); By using random-effects meta-analysis, the pooled prevalence of any RVO was 0.95% (95% CI=0.75-1.22) (**Figure S1**).

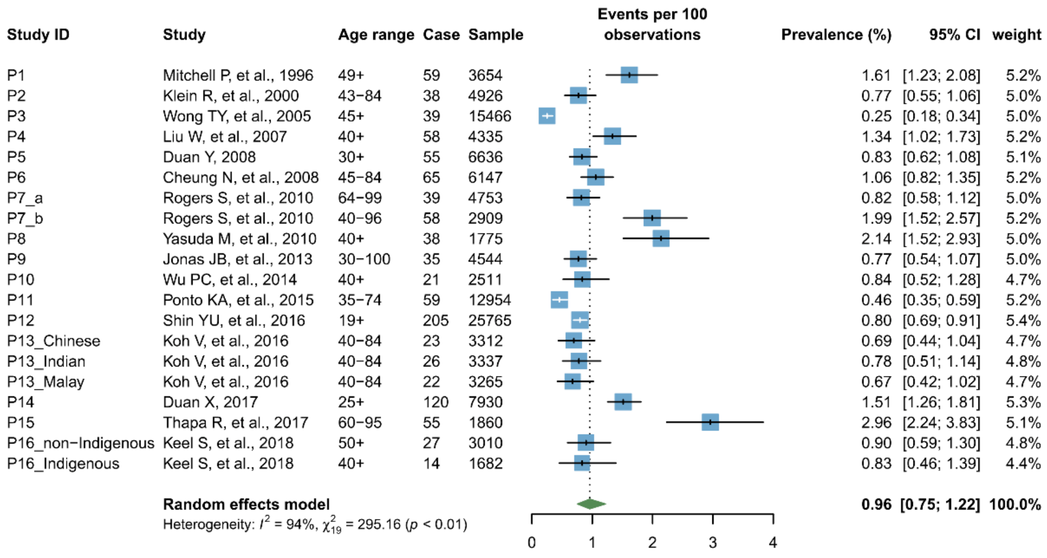

Figure S1. Forest plot of studies reporting the prevalence of any RVO (n=20)

The sensitivity analysis found that the pooled prevalence of any RVO varied from 0.90% (95% CI=0.72-1.13) to 1.03% (95% CI=0.83-1.28) after removing one study at one time, no single study had significantly influenced the liability and stability of the overall pooled prevalence (**Figure S2**).

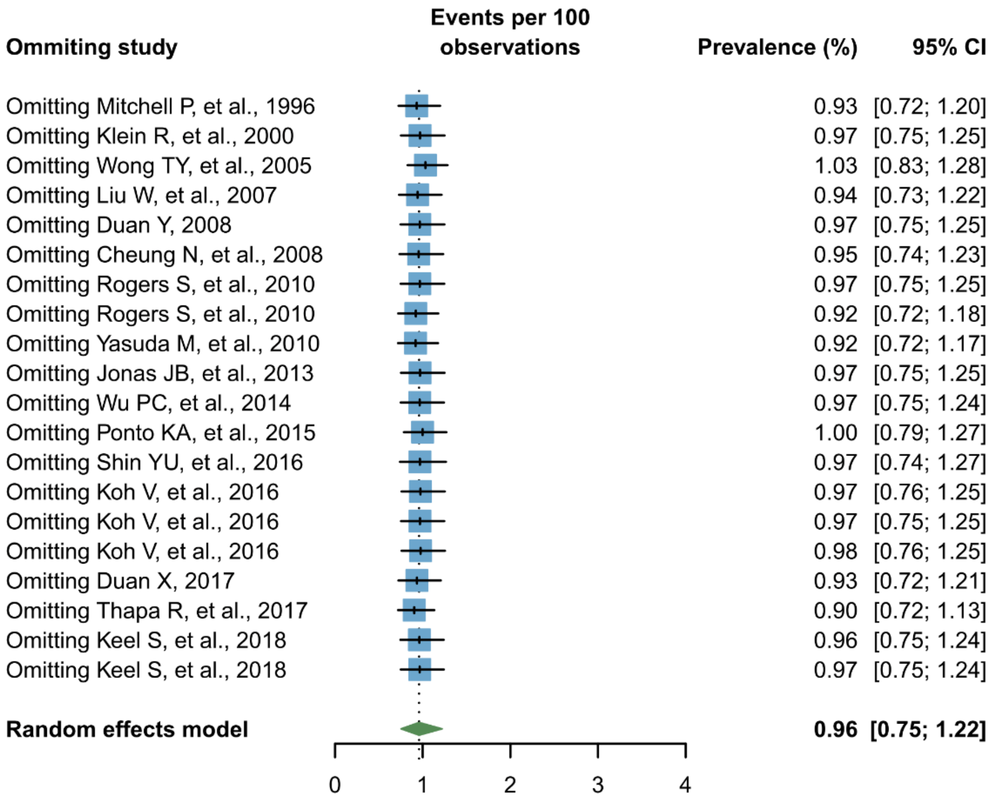

Figure S2. Leave-one-out sensitivity analysis of the influence of single study on the pooled prevalence of any RVO (n=20)

According to funnel plot, Egger's test (t=-0.500, p= 0.623) and Begg's test (z= -0.714, p= 0.475), no publication bias was revealed (**Figure S3**).

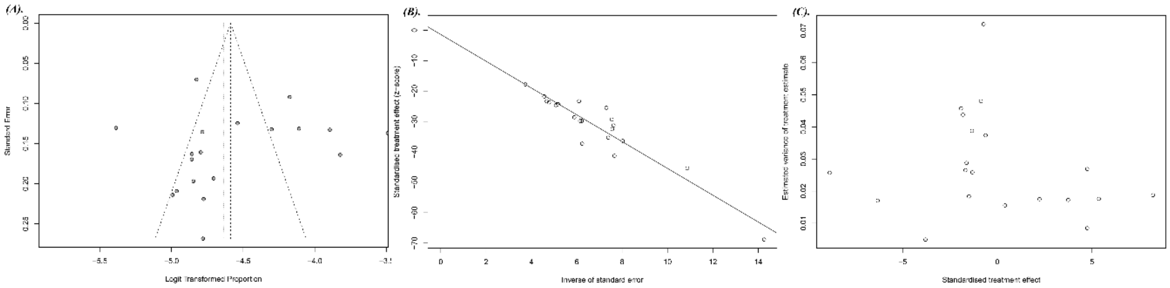

Figure S3. Publication bias of studies reporting the prevalence of any RVO (n=20)
Note: (A) Funnel plot; (B) Egger's test; (C) Begg's test.

Table S6. Meta-analyses of studies on any BRVO prevalence

Significantly high heterogeneity also existed between studies that reported the prevalence of BRVO (I^2 =93.1%, p<0.001). Therefore, a random-effects meta-analysis was adopted, where a pooled prevalence of BRVO of 0.79% (95% CI=0.60-1.04) was revealed (**Figure S4**).

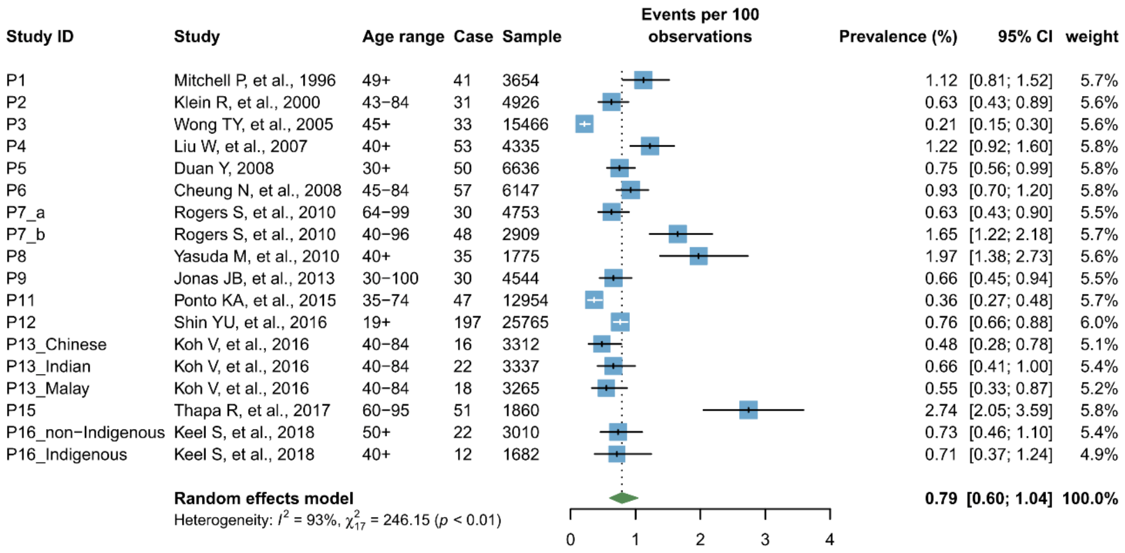

Figure S4. Forest plot of studies reporting the prevalence of BRVO (n=18)

When removing one single study at one time, the pooled prevalence of BRVO varied from 0.73% (95% CI=0.58-0.94) to 0.86% (95% CI=0.67-1.10). No single study significantly influenced the liability and stability of the pooled prevalence of BRVO (**Figure S5**).

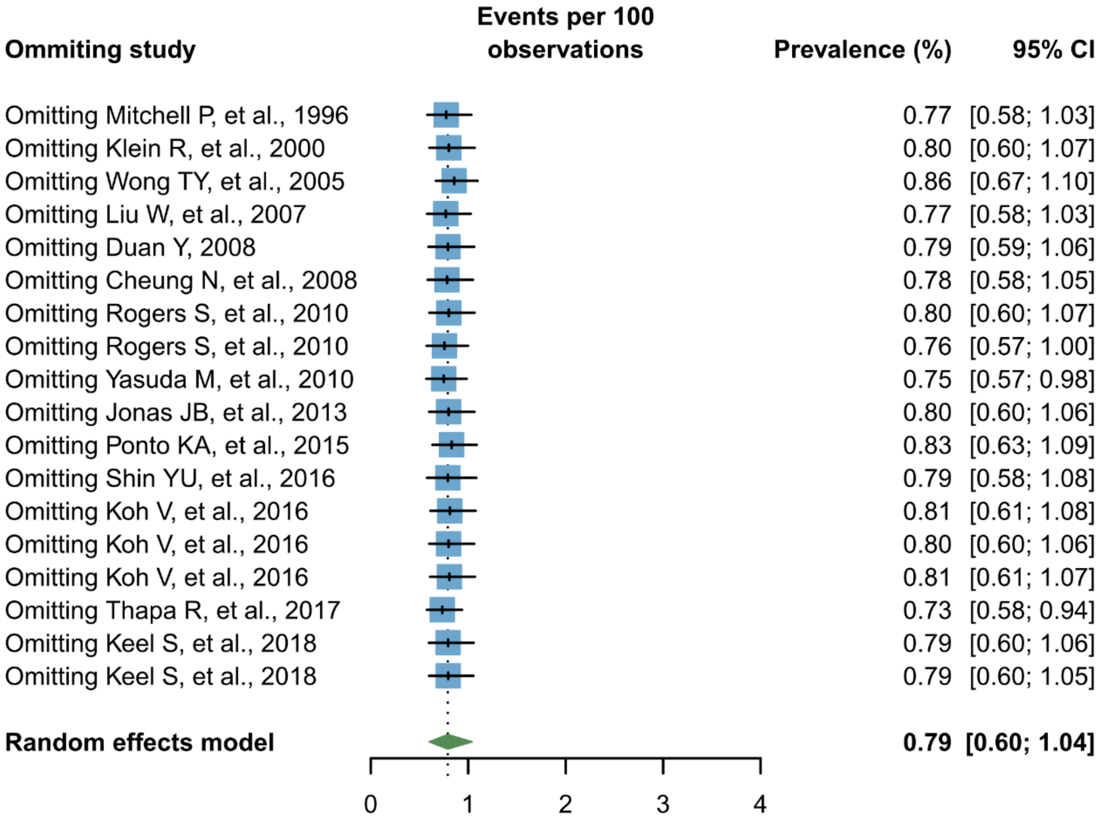

Figure S5. Leave-one-out sensitivity analysis of the influence of single study on the pooled

prevalence of BRVO (n=18)

Visually inspection of the funnel plot didn’t suggest potential publication bias. Moreover, neither Egger’s test (t= -0.517, p= 0.612) or Begg’s test (z= -1.099, p= 0.272) significantly indicated any publication bias (**Figure S6**).

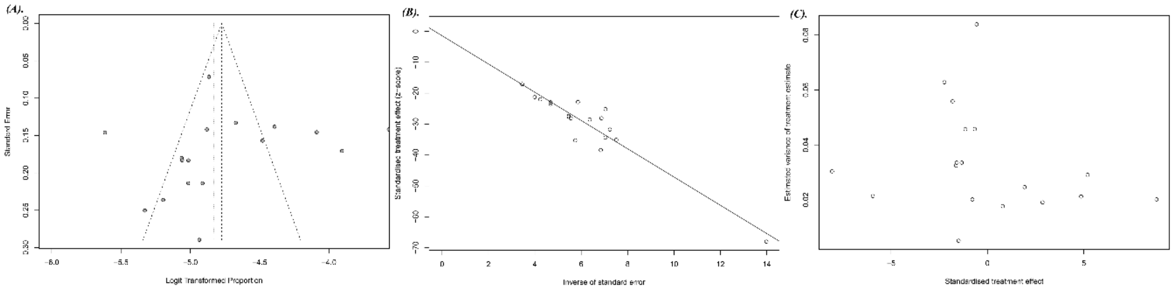

Figure S6. Publication bias of studies reporting the prevalence of BRVO (n=18)
Note: (A) Funnel plot; (B) Egger’s test; (C) Begg’s test.

Table S7. Meta-analyses of studies on any CRVO prevalence

Similarly, moderate heterogeneity was also significantly detected between studies reporting the prevalence of CRVO (I^2 =71.8%, p<0.001). The random-effects meta-analysis revealed the pooled prevalence of CRVO as 0.13% (95% CI=0.09-0.19) (**Figure S7**).

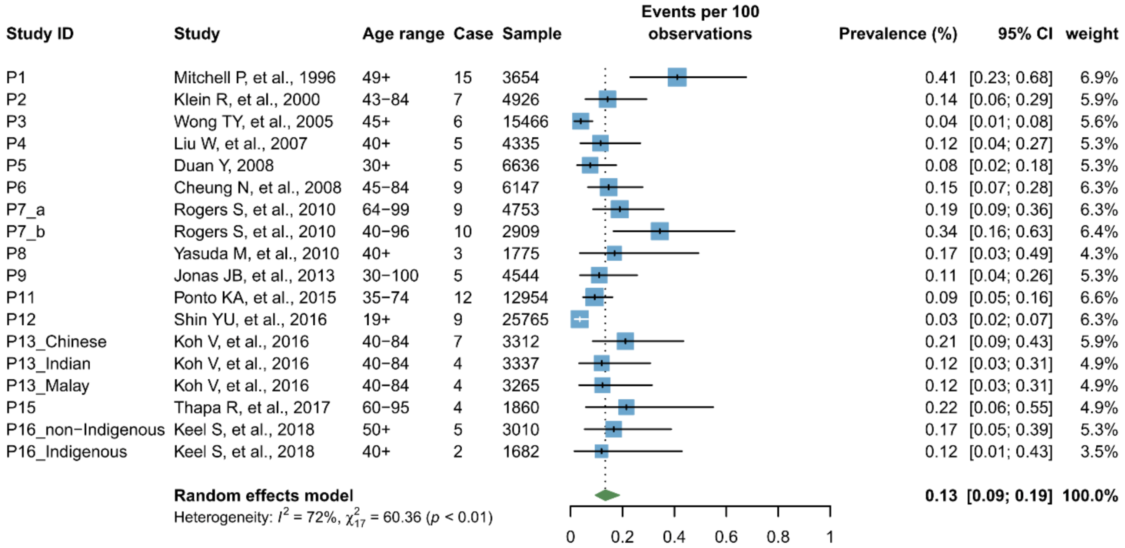

Figure S7. Forest plot of studies reporting the prevalence of CRVO (n=18)

According to the leave-one-out sensitivity analysis, the pooled prevalence of CRVO ranged from 0.12% (95% CI=0.09-0.17) to 0.15% (95% CI=0.11-0.20), the liability and stability of meta-analysis were not significantly influenced by any single studies (**Figure S8**).

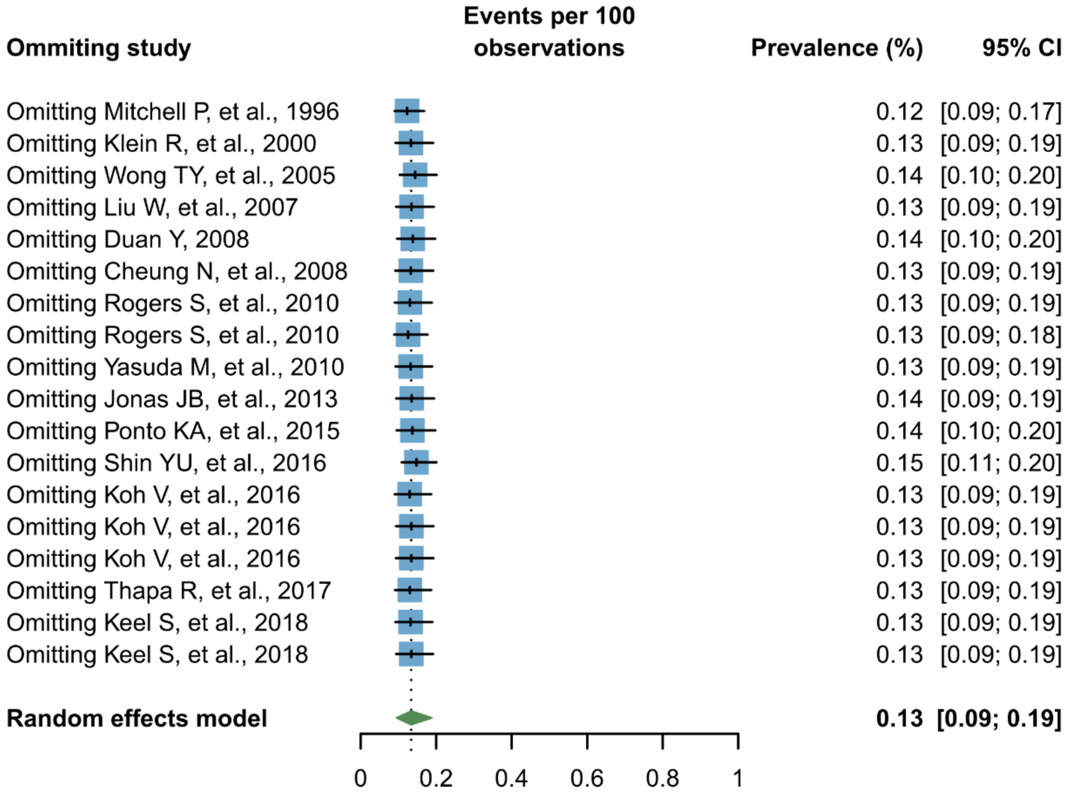

Figure S8. Leave-one-out sensitivity analysis of the influence of single study on the pooled

prevalence of CRVO (n=18)
Visual inspection of funnel plot, Egger’s test (t= -1.095, p= 0.290) or Begg’s test (z= -0.038, p= 0.970) didn’t reveal a risk of publication bias (**Figure S9**).

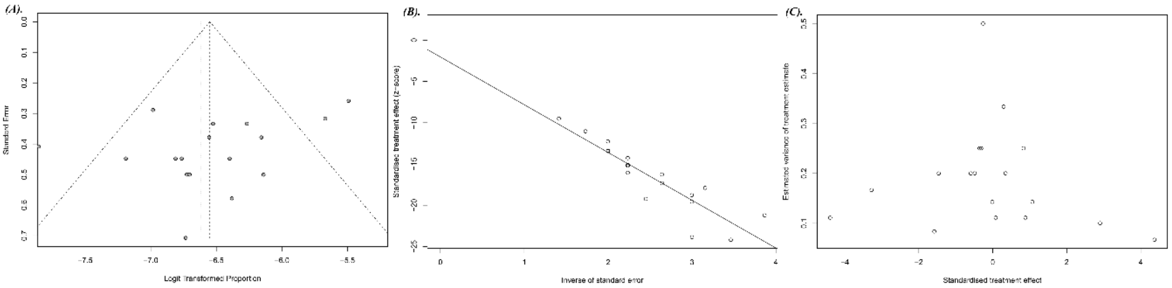

Figure S9. Publication bias of studies reporting the prevalence of CRVO (n=18)
Note: (A) Funnel plot; (B) Egger’s test; (C) Begg’s test.

Table S8. Meta-analyses of risk factors for any RVO

Risk factor 1-Advanced age (per decade)

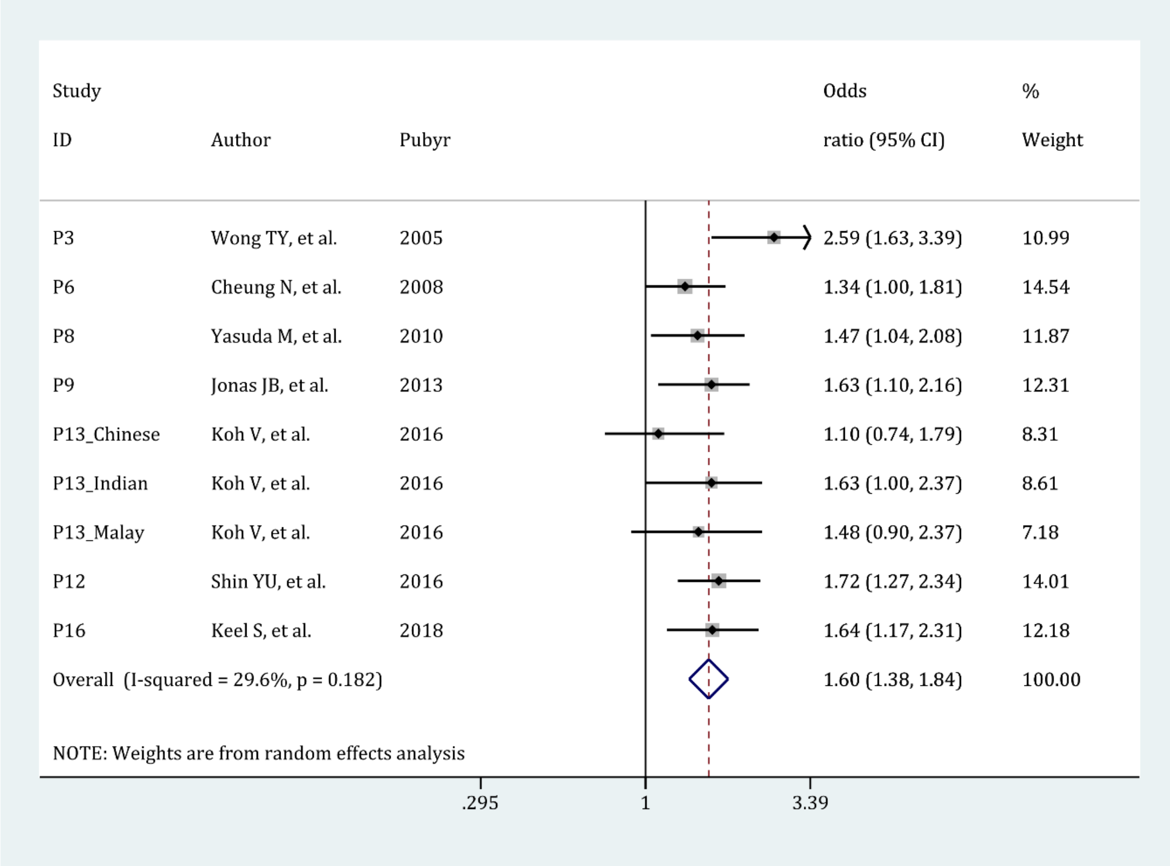

Risk factor 2-Female sex

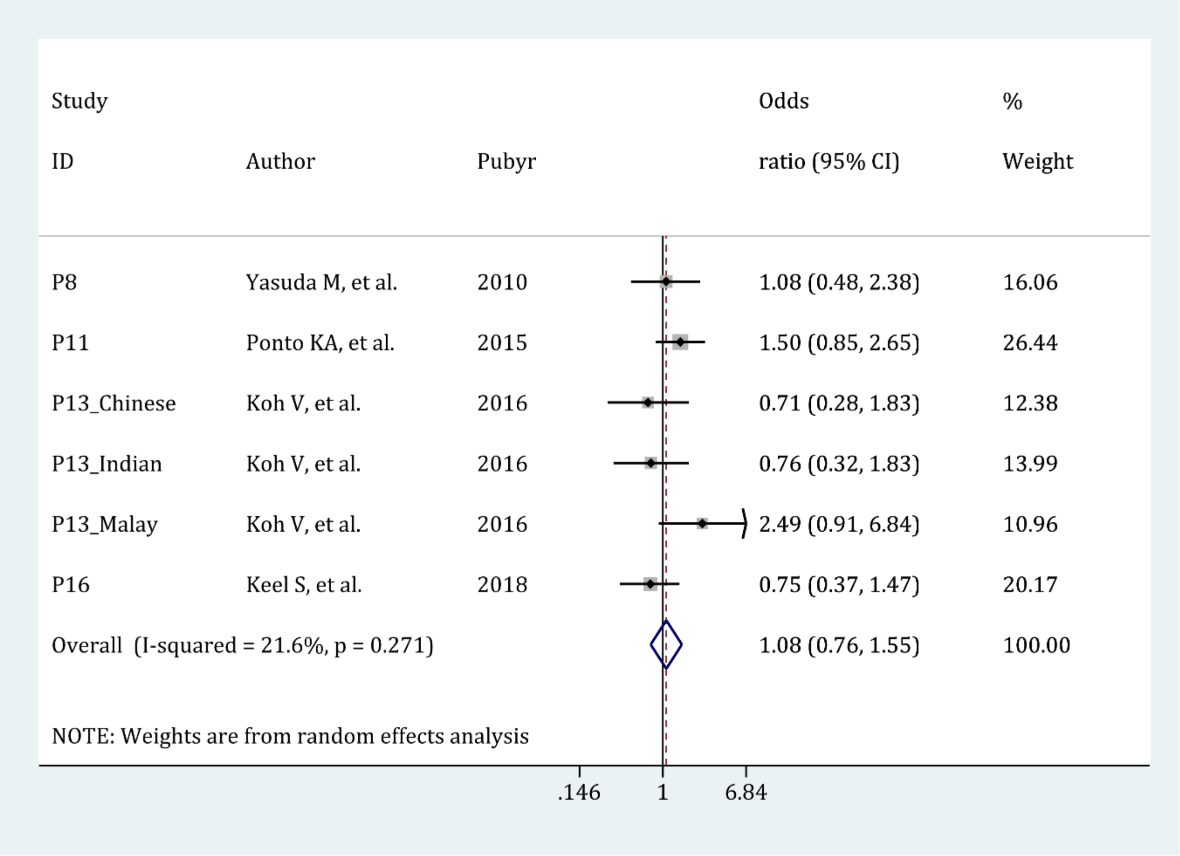

Risk factor 3-Creatinine (per 10 mmol/L increase)

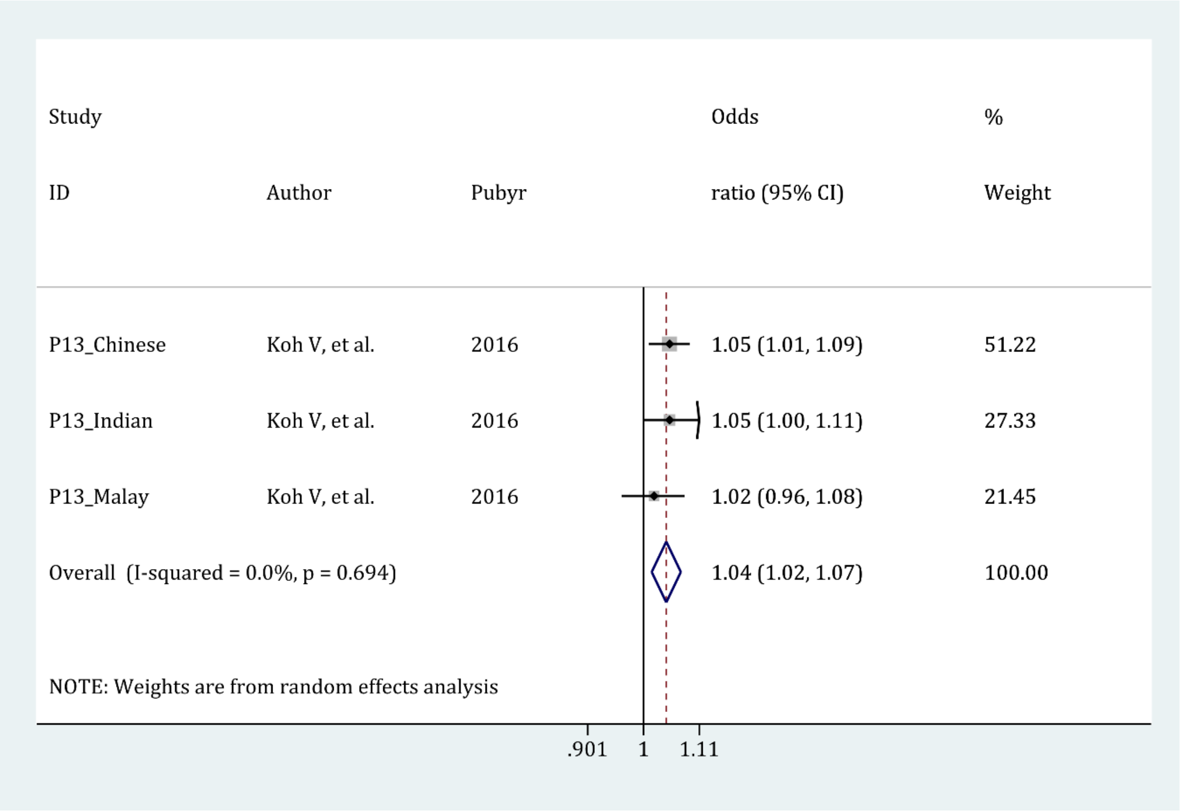

Risk factor 4-Vertical cup-to-disc ratio (per 1.0 increase)

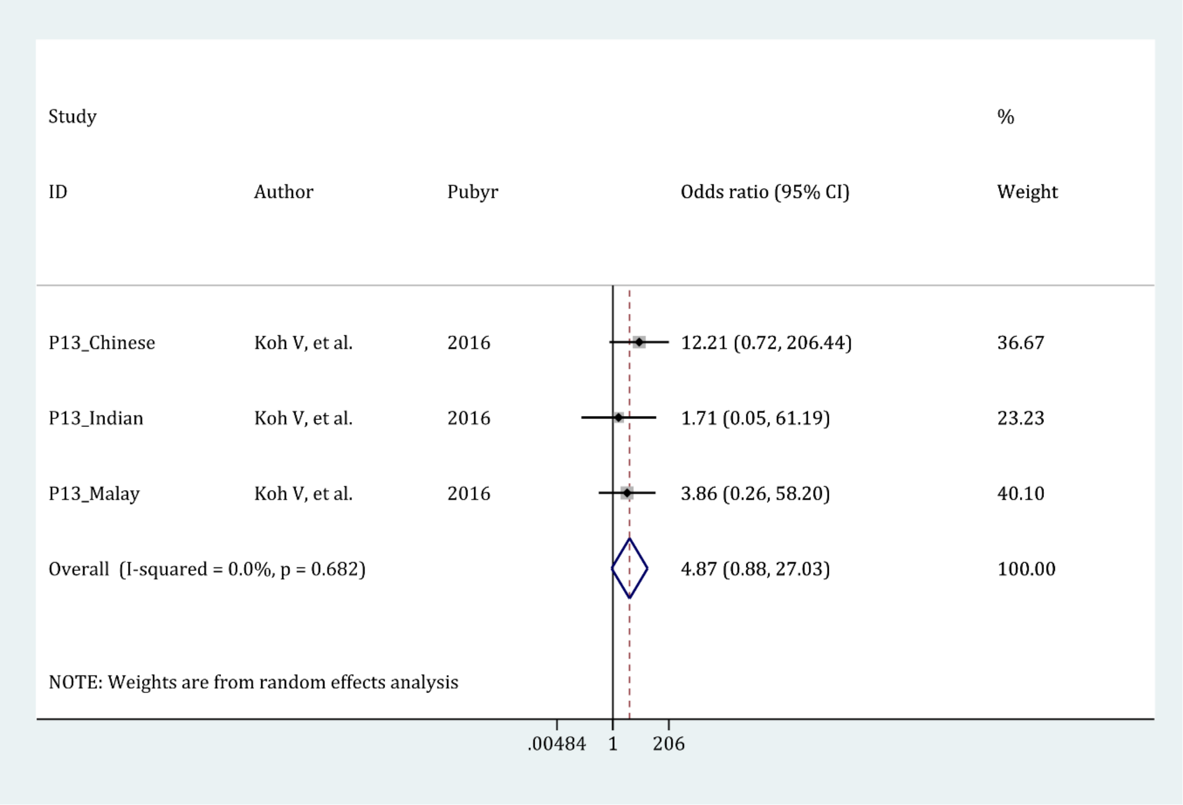

Risk factor 5-Heart Attack

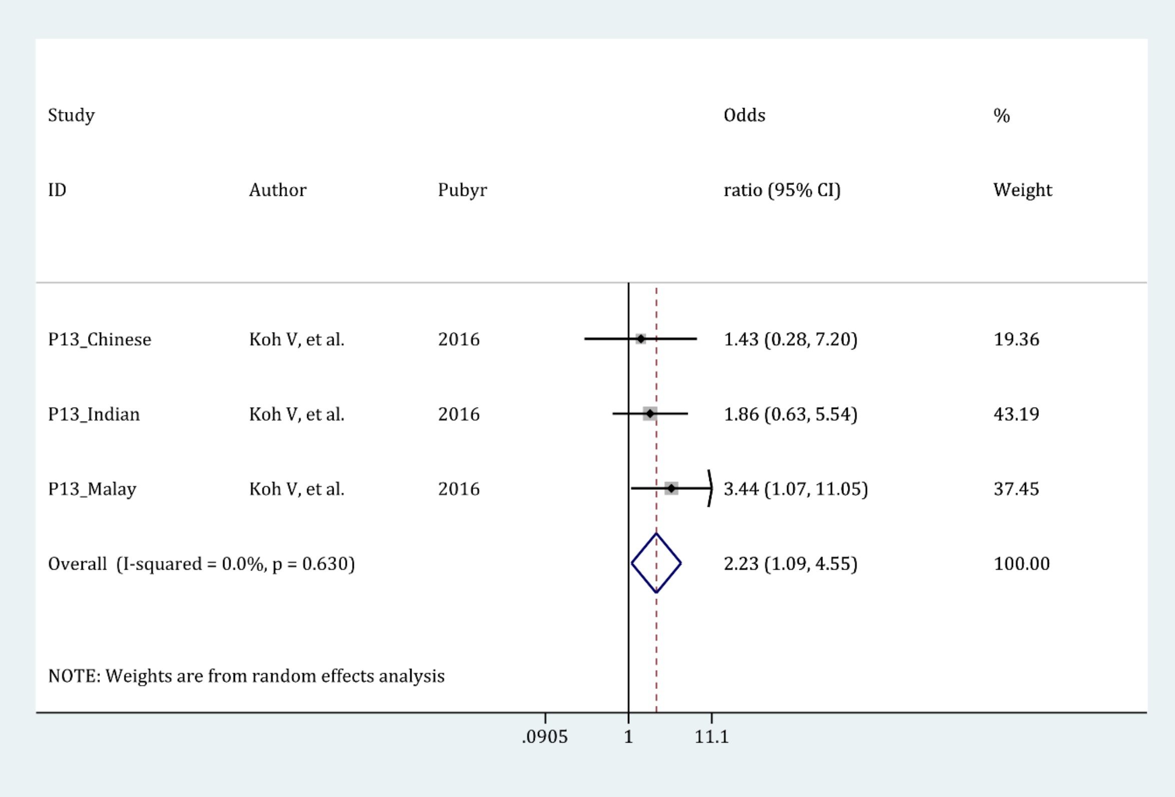

Risk factor 6-Total cholesterol (per mmol/L)

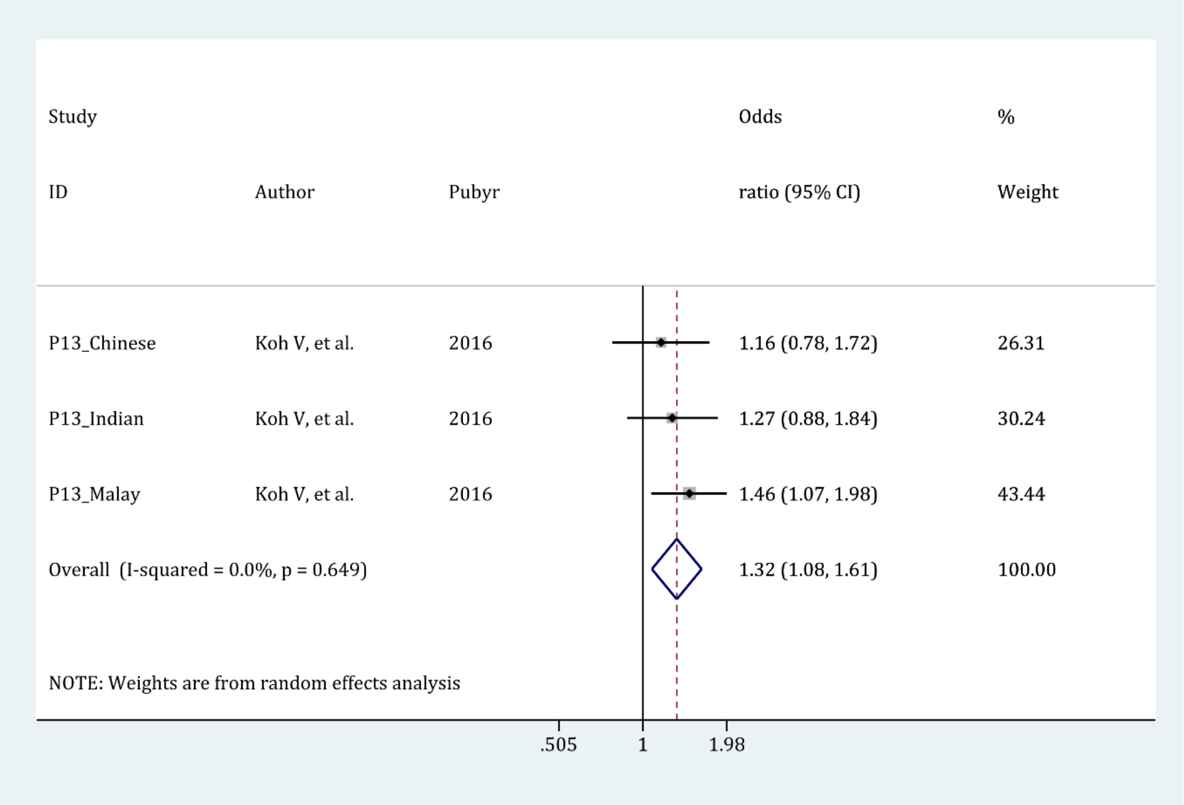

Risk factor 7-Diabetes

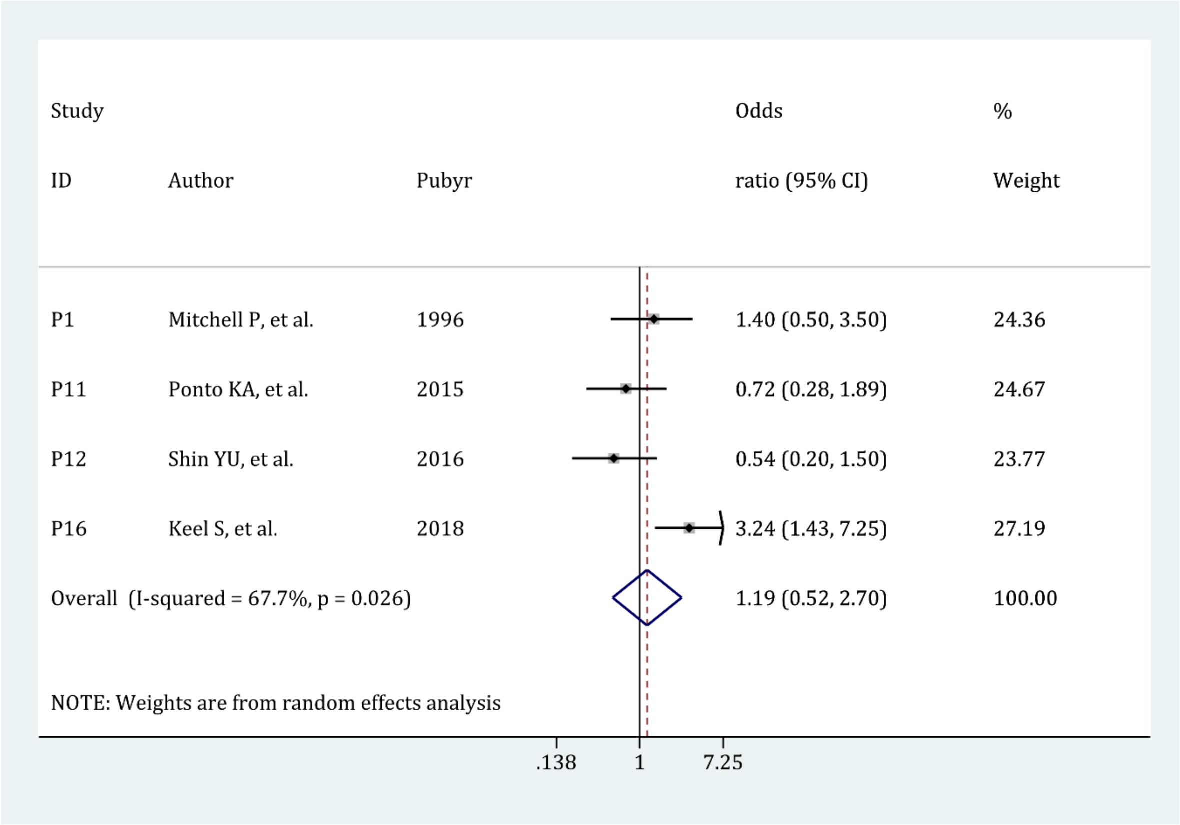

Risk factor 8-Stroke

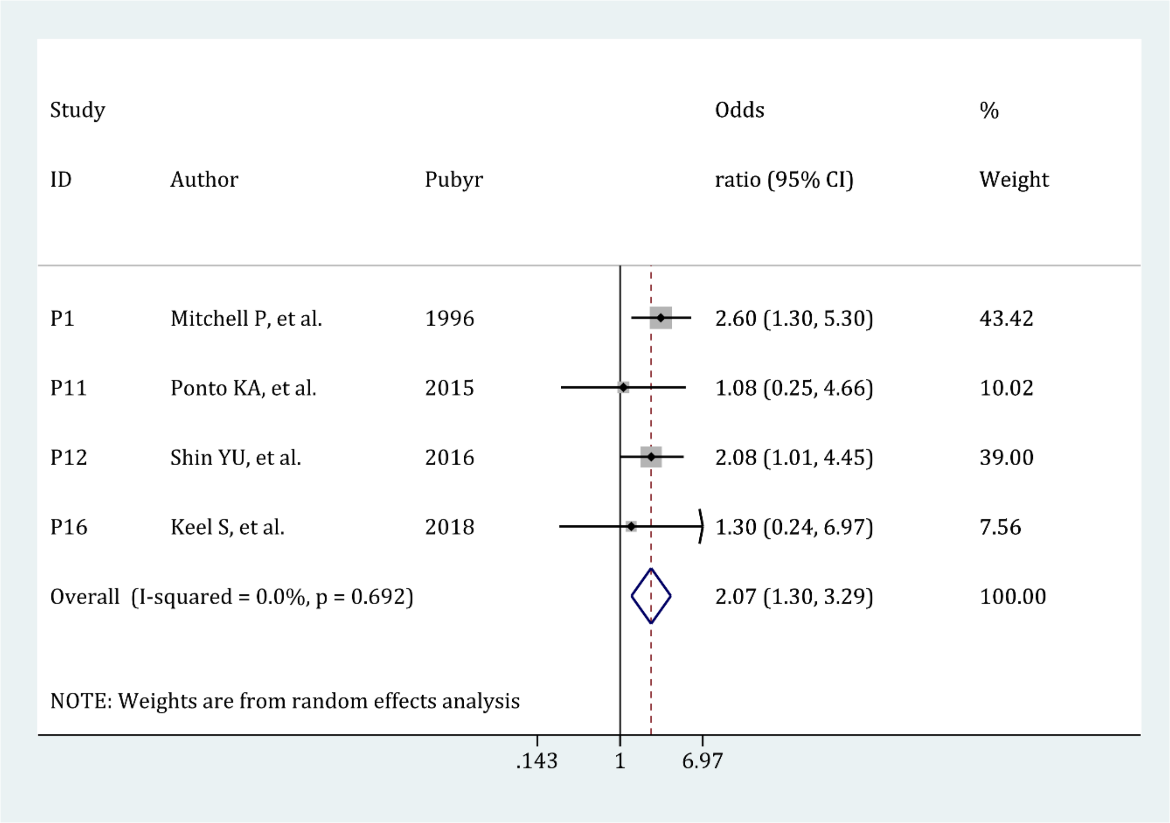

Risk factor 9-Hypertension

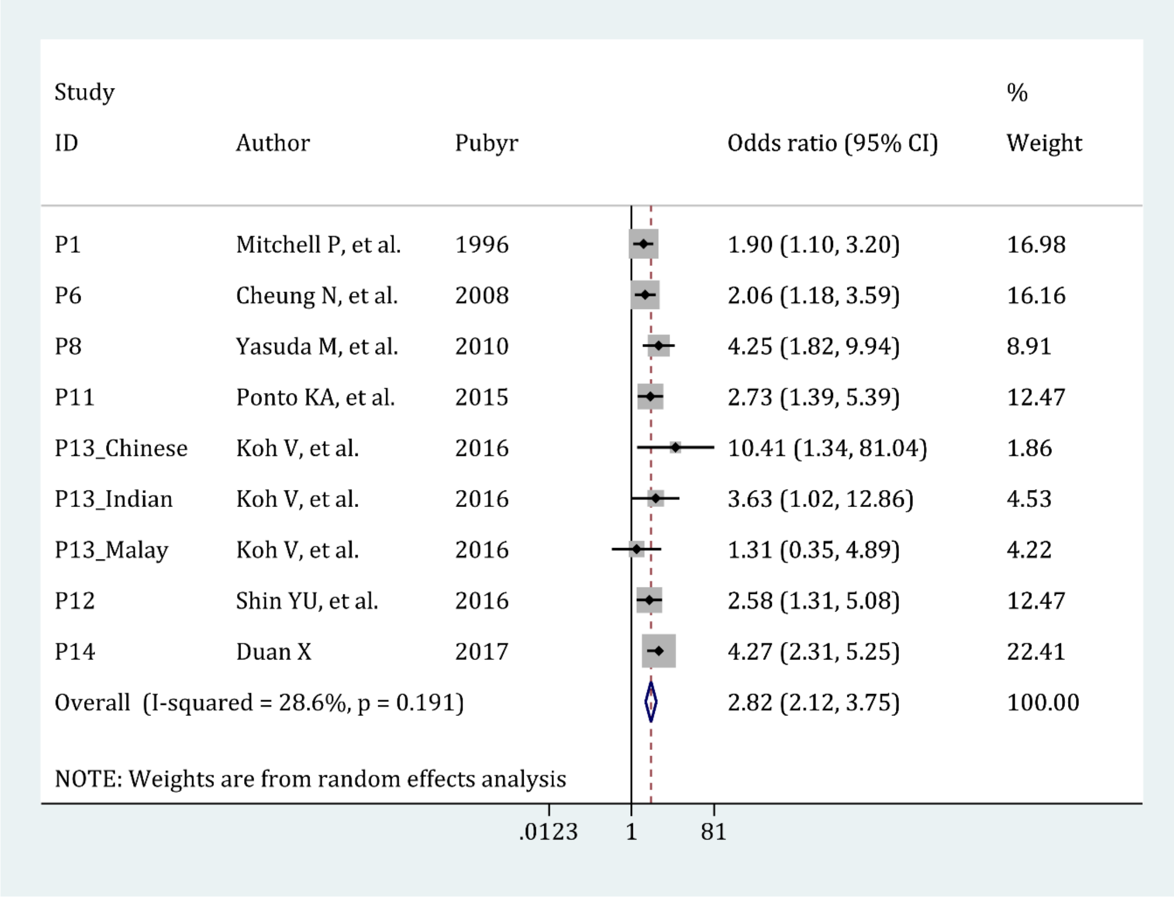

References

1 Mitchell P, Smith W, Chang A. Prevalence and associations of retinal vein occlusion in Australia. The Blue Mountains Eye Study. Arch Ophthalmol. 1996;114:1243-7.

2 Klein R, Klein BE, Moss SE, Meuer SM. The epidemiology of retinal vein occlusion: the Beaver Dam Eye Study. Trans Am Ophthalmol Soc. 2000;98:133-41.

3 Wong TY, Larsen EK, Klein R, Mitchell P, Couper DJ, Klein BE, et al. Cardiovascular risk factors for retinal vein occlusion and arteriolar emboli: the Atherosclerosis Risk in Communities & Cardiovascular Health studies. Ophthalmology. 2005;112:540-7.

4 Weiwei L, Xu L, Xie X, Peng X, Yang H, Li J. EpidemioIogy of retinaI vein occlusion in adult Chinese population in rural and urban areas in Beijing (北京城乡人群视网膜静脉阻塞患病情况调查). Ophthalmol CHN (眼科). 2007:233-6.

5 Duan Y. The prevelence of retinal vein occlusion and analysis of its related risk factors in rural area of North China (中国北方农村地区视网膜静脉阻塞患病率调查及相关危险因素分析) [Master Dissertation]: Central South University (中南大学); 2008.

6 Cheung N, Klein R, Jie JW, Cotch MF, Islam AFM, Klein BEK, et al. Traditional and novel cardiovascular risk factors for retinal vein occlusion: The multiethnic study of atherosclerosis. Invest Ophthalmol Vis Sci. 2008;49:4297-302.

7 Rogers S, McIntosh RL, Cheung N, Lim L, Wang J, Mitchell P, et al. The prevalence of retinal vein occlusion: pooled data from population studies from the United States, Europe, Asia, and Australia. Ophthalmology. 2010;117:313-e1.

8 Augood C, Fletcher A, Bentham G, Chakravarthy U, De Jong P, Rahu M, et al. Methods for a population-based study of the prevalence of and risk factors for age-related maculopathy and macular degeneration in elderly European populations: the EUREYE study. Ophthalmic Epidemiol. 2004;11:117-29.

9 West SK, Klein R, Rodriguez J, Muñoz B, Broman AT, Sanchez R, et al. Diabetes and diabetic retinopathy in a Mexican-American population: Proyecto VER. Diabetes Care. 2001;24:1204-9.

10 Yasuda M, Kiyohara Y, Arakawa S, Hata Y, Yonemoto K, Doi Y, et al. Prevalence and systemic risk factors for retinal vein occlusion in a general Japanese population: the Hisayama study. Invest Ophthalmol Vis Sci. 2010;51:3205-9.

11 Jonas J, Nangia V, Khare A, Sinha A, Lambat S. Prevalence and associations of retinal vein occlusions: The Central India Eye and Medical Study. Retina. 2013;33:152-9.

12 Wu P, Zhang W, Lu P, Chen S, Tao M. Epidemical survey of relative factors of retinal vessels disease of the native Tibetan among the people aged 40 and above in Maqin county, Qinghai province (青海省玛沁县 40 岁以上世居藏族人群视网膜血管性疾病相关因素的流行病学调查). Int Eye Sci (国际眼科杂志). 2014:1288-91.

13 Ponto KA, Elbaz H, Peto T, Laubert-Reh D, Binder H, Wild PS, et al. Prevalence and risk factors of retinal vein occlusion: the Gutenberg Health Study. J Thromb Haemost. 2015;13:1254-63.

14 Shin YU, Cho H, Kim JM, Bae K, Kang MH, Shin JP, et al. Prevalence and associated factors of retinal vein occlusion in the Korean National Health and Nutritional Examination Survey, 2008-2012: A cross-sectional observational study. Medicine. 2016;95:e5185.

15 Koh V, Cheung CY, Li X, Tian D, Wang J, Mitchelle P, et al. Retinal vein occlusion in a multi-ethnic Asian population: the Singapore Epidemiology of Eye Disease Study. Ophthalmic Epidemiol. 2016;23:6-13.

16 Duan X. Investigation of the risk factors of retinal vein occlusion in Qingyang (庆阳市居民视网膜静脉阻塞发病情况及危险因素调查). Chin J of PHM (中国公共卫生管理). 2017:84-5+8.

17 Thapa R, Bajimaya S, Paudyal G, Khanal S, Tan S, Thapa SS, et al. Prevalence, pattern and risk factors of retinal vein occlusion in an elderly population in Nepal: the Bhaktapur retina study. BMC Ophthalmol. 2017;17:162.

18 Keel S, Xie J, Foreman J. Prevalence of retinal vein occlusion in the Australian National Eye Health Survey. Clin Exp Ophthalmol. 2018;46:260-5.

19 Cugati S, Jie JW, Rochtchina E, Mitchell P. Ten-year incidence of retinal vein occlusion in an older population: The blue mountains eye study. Arch Ophthalmol. 2006;124:726-32.
20 Klein BE, Meuer SM, Knudtson MD, Klein R. The relationship of optic disk cupping to retinal vein occlusion: the Beaver Dam Eye Study. Am J Ophthalmol. 2006;141:859-62.
21 Arakawa S, Yasuda M, Nagata M, Ninomiya T, Hirakawa Y, Doi Y, et al. Nine-year incidence and risk factors for retinal vein occlusion in a general Japanese population: the Hisayama Study. Invest Ophthalmol Vis Sci. 2011;52:5905-9.
22 Klein R, Moss SE, Meuer SM, Klein BE. The 15-year cumulative incidence of retinal vein occlusion: the Beaver Dam Eye Study. Arch Ophthalmol. 2008;126:513-8.
23 Zhou J, Xu L, Wang S, Wang Y, You Q, Tu Y, et al. The 10-year incidence and risk factors of retinal vein occlusion: the Beijing Eye Study. Ophthalmology. 2013;120:803-8.
